# S1P_1_ deletion differentially affects TH17 and Regulatory T cells

**DOI:** 10.1038/s41598-017-13376-2

**Published:** 2017-10-10

**Authors:** Ahmet Eken, Rebekka Duhen, Akhilesh K. Singh, Mallory Fry, Jane H. Buckner, Mariko Kita, Estelle Bettelli, Mohamed Oukka

**Affiliations:** 1 Seattle Children’s Research Institute, Center for Immunity and Immunotherapies, Seattle, WA 98101 USA; 2 Medical Biology Department, Genome and Stem Cell Center (Genkok), Faculty of Medicine, Erciyes University, Melikgazi, Kayseri, 38039 Turkey; 3 Benaroya Research Institute at Virginia Mason, Seattle, WA 98101 USA; 4 University of Washington, Department of Immunology, Seattle, WA 98105 USA

## Abstract

Sphingosine-1 phosphate receptor 1 (S1P_1_) is critical for the egress of T and B cells out of lymphoid organs. Although S1P_1_ agonist fingolimod is currently used for the treatment of multiple sclerosis (MS) little is known how S1P_1_ signaling regulates Th17 and T_reg_ cell homeostasis. To study the impact of S1P_1_ signaling on Th17 and T_reg_ cell biology, we specifically deleted S1P_1_ in Th17 and T_reg_ cells using *IL-*1*7A* ^*Cre*^ and *Foxp3* ^*Cre*^ mice, respectively. Deletion of S1P_1_ in Th17 cells conferred resistance to experimental autoimmune encephalomyelitis (EAE). On the other hand, permanent deletion of S1P_1_ in T_reg_ cells resulted in autoimmunity and acute deletion rendered mice more susceptible to EAE. Importantly, our study revealed that S1P_1_ not only regulated the egress of T_reg_ cells out of lymphoid organs and subsequent non-lymphoid tissue distribution but also their phenotypic diversity. Most of the T_reg_ cells found in S1P_1_-deficient mice as well as MS patients on fingolimod therapy had an activated phenotype and were more prone to apoptosis, thus converted to effector T_reg_. Our results provide novel insight into the functions of S1P_1_ and potential impact of long term fingolimod use on Th17 and T_reg_ cell biology and general health in MS patients.

## Introduction

Sphingosine 1 phosphate receptor 1 (S1P_1_) is a G-protein coupled receptor expressed by endothelial cells and lymphocytes, including T_reg_ cells. S1P_1_ activates various signaling cascades, including PI3K-Akt-mTOR upon binding to its natural ligand sphingosine-1 phosphate (S1P)^1^. S1P_1_ was previously shown to play a critical role in the egress of both T and B cells out of thymus and lymphoid organs^2–4^. A gradient of S1P which is high in blood and lymph, and low in tissues, is created by tight regulation of its production^5,6^. This gradient of S1P coupled with ligand binding-triggered receptor internalization forms the basis of the egress mechanism for T and B cells^7^. Fingolimod (FTY720 or Gilenya^TM^) is a structural analog of sphingosine-1; upon binding to S1P_1_, it induces its internalization and desensitization, thereby causing sequestration of lymphocytes in lymphoid tissues^8^. Although approved for the treatment of multiple sclerosis^9^, in some patients, cessation or initiation of fingolimod therapy resulted in exacerbation of MS and/or formation of tumefactive lesions in the brain through yet unexplored mechanisms^10–14^.

Th17 cells are required for the pathogenesis of multiple autoimmune and chronic inflammatory conditions, including EAE, a murine model of MS. Although S1P_1_ was genetically targeted broadly in all CD4^+^ T cells previously, T helper lineage specific knockout murine models of S1P_1_ have not been studied, thus, it is unknown how S1P_1_ or fingolimod modulates the biology of Th17 lineage independently of its effects on other helper T cell lineages. CD4^+^Foxp3^+^ regulatory T cells (T_reg)_, on the other hand, are crucial for preventing autoimmunity and restraining effector T cell responses during protective immunity^15,16^. Similarly, the role of S1P_1_ in exclusively committed T_reg_ cell homeostasis has been less clear, as the mice used in previous reports had deleted S1P_1_ in all CD4^+^ T cells.

Recent studies revealed that non-lymphoid tissue (NLT) resident T_reg_ cells assume different phenotypic features than those in circulation or lymphoid tissues (LT)^16,17^. NLT T_reg_ cells resemble conventional effector CD4^+^ T cells, and express high levels of CD44, low levels of CD62L and CCR7 and are named effector T_reg_ (eT_reg)_^18^. eT_reg_ cells also express CD103, KLRG1 and ICOS. eT_reg_ cells were shown to be dependent on ICOSL stimulation provided by antigen presenting cells (APC) for their homeostasis in tissue microenvironments lacking IL-2 and appear to be more prone to apoptosis^19^. In contrast, LT or circulatory T_reg_ cells inversely express the above-mentioned molecules. They are named central T_reg_ (cT_reg)_ and, conversely, cT_reg_ cells rely more on IL-2 than ICOS for their homeostasis and are resistant to apoptosis^19^. This dichotomous phenotypic subdivision of murine T_reg_ and survival mechanisms are also valid for human T_reg_ cells^20^. Human cT_reg_ cells can be defined as CD4^+^CD45RA^+^CD45RO^−^CD25^+^CD127^−^Foxp3^low^. Conversely, human CD4^+^Foxp3^+^ eT_reg_ cells are CD45RA^−^CD45RO^+^CD25^high^CD127^−^Foxp3^high^. More recently, C-C chemokine receptor 4 (CCR4) was defined as a marker of human eT_reg_ along with other effector non-T_reg_ T cells, and was targeted for depletion of exclusively eT_reg_ cell populations^21^. The studies using broad deletion of S1P_1_ in T cells (using the CD4^cre^ system) showed improved T_reg_ generation and function in the absence of this receptor^22^. In contrast, S1P_1_ overexpression in CD4 T^+^ cells reduced their differentiation into T_reg_ cells and functions through PI3K-Akt-mTOR axis and its effect on Smad3 transcription factor^22,23^. However, in these studies S1P_1_ deletion was not unique to T_reg_ cells. More importantly, it remains unknown how S1P_1_ regulates function and egress of specifically committed T_reg_ cells.

By permanent and/or temporal genetic deletion of S1P_1_, herein we show that S1P_1_ regulates proper Th17 and T_reg_ cell distribution across peripheral organs and homing to the central nervous system and their functions as well as EAE development in mice. We also show that S1P_1_ regulates phenotypic diversity of murine and human T_reg_ cells by controlling central to effector T_reg_ cell switch. Our data provides novel insights into the egress-dependent and independent functions of S1P_1_ and potential impact of long term fingolimod use on T_reg_ cell homeostasis.

## Results

### S1P_1_ regulates generation and peripheral distribution of Th17 cells and susceptibility to EAE

**Figure 1:**
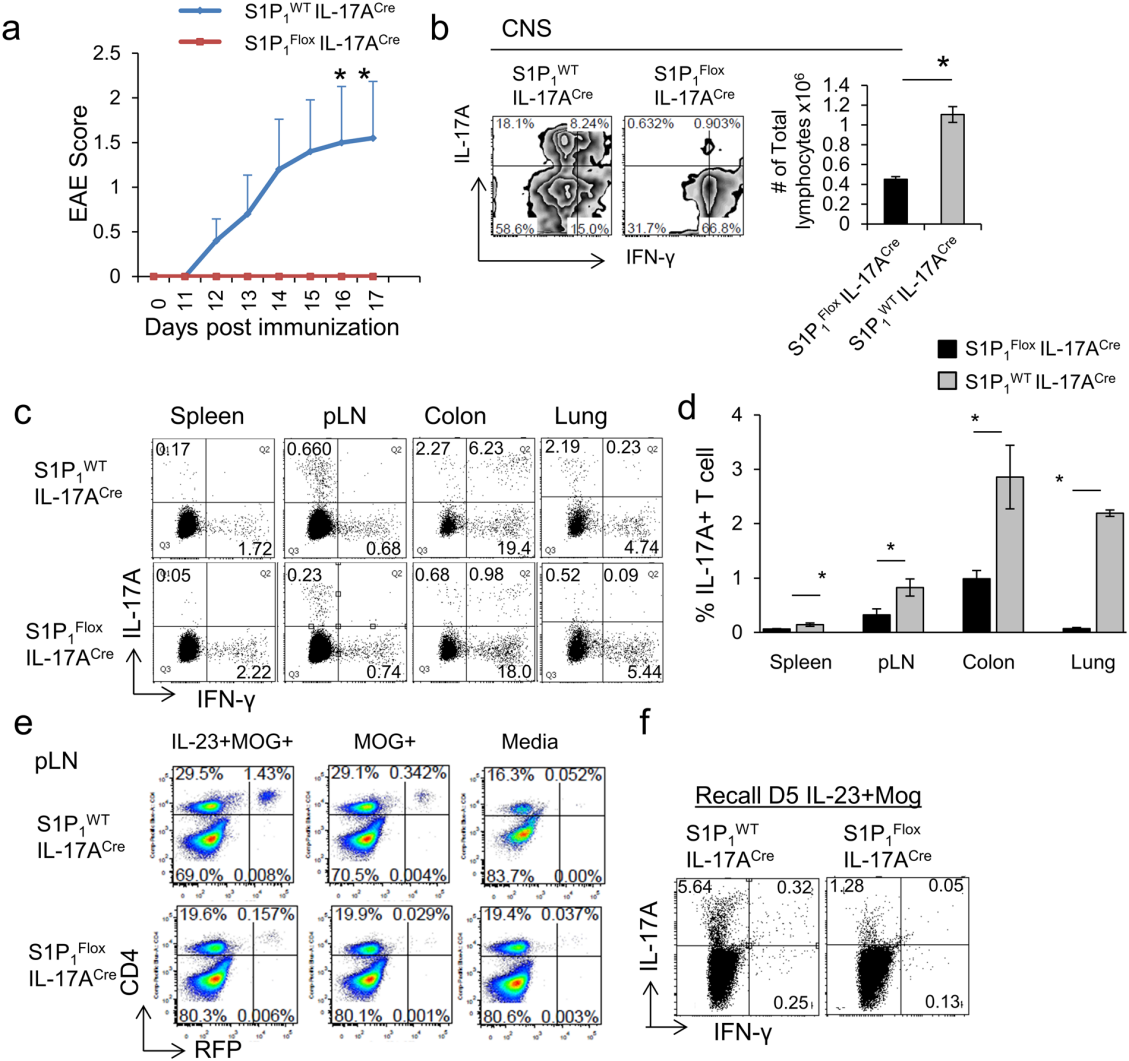
S1P_1_ is required for peripheral organ distribution and *in vivo* generation of Th17 cells. (**a**) EAE scores of *S1P*_*1*_^*Flox*^ *IL-17A* ^*Cre*^ *ROSA* ^*RFP*^and control mice (*S1P*_*1*_^*WT*^ *IL-17A* ^*Cre*^ *ROSA* ^*RFP*^) that were immunized with MOG_35–55_ and Complete Freund’s Adjuvant. N = 5 mice per group, the experiment was repeated three times. (**b**) IL-17A and IFN-γ expression of CD4+ T cells from central nervous system (CNS) of *S1P*_*1*_^*Flox*^ *IL-17A* ^*Cre*^ *ROSA* ^*RFP*^ and control mice 15 days after MOG_35–55_ immunization (*left*). The cells were cultured 4 h with PMA/ionomycin and Golgi Plug for intracellular staining. Quantification of flow plots, (*right*).* indicates p-value <0.05. (**c**) Th17 and Th1 cell distribution across the peripheral organs in *S1P*_*1*_^*Flox*^ *IL-17A* ^*Cre*^ *ROSA* ^*RFP*^ and control mice, 7 days after MOG_35–55_ immunization, and quantification was shown in (**d**). (**e**) Representative flow plot of RFP^+^ CD4^+^ cells after rechallenge with media, MOG_35–55_ or MOG_35–55_ + IL-23 following the first immunization. (**f**) IL-17A and IFN-γ production by *S1P*_*1*_^*Flox*^ *IL-17A* ^*Cre*^ *ROSA* ^*RFP*^ and control mice in MOG_35–55_ + IL-23 rechallenge condition.

To uncover the role of S1P_1_ on Th17 cell biology we crossed *S1P*_*1*_^*Flox*^ to *IL-*1*7A* ^*Cre*^ *ROSA* ^*RFP*^ mice. Red fluorescent reporter (RFP) allowed us to gate on all the IL-17A expressing T cells when necessary (including true-Th17 and ex-Th17 which are RFP^+^ but no longer expressing IL-17A). Deletion of S1P_1_ from Th17 cells rendered these mice completely resistant to the development of EAE induced by MOG_35–55_ immunization (Fig. 1a). There were significantly fewer infiltrating leukocytes, including Th17 and Th1 cells, in the central nervous system (CNS) of *S1P*_*1*_^*Flox*^ *IL-*1*7A* ^*Cre*^ mice compared with controls (Fig. 1b). These results are in line with previous findings that S1P_1_ regulates T cell egress out of lymphoid organs, thus they are unable to home to CNS during EAE (21–24). To ensure that the past observations on CD4+ T cells are also applicable specifically to Th17 lineage, we examined Th17 cell distribution across the peripheral organs in *S1P*_*1*_^*Flox*^ *IL-*1*7A* ^*Cre*^ *ROSA* ^*RFP*^ and control mice, before (not shown) and after MOG_35–55_ immunization (Fig. 1c,d). As expected, we observed a significant reduction in the percentages of Th17 cells in the lung, spleen and colon (Fig. 1c,d). Surprisingly, Th17 cell ratio in the lymph nodes also decreased, even after immunization, in contrast to the proposed role of S1P_1_ for egress of T cells out of lymph nodes (LN) and the expected entrapment of these cells. These results suggest that Th17 generation and/or survival may be regulated by S1P_1_. We did not see a conclusive difference in the apoptosis of peripheral LN Th17 cells between control and *S1P*_*1*_^*Flox*^ *IL-*1*7A* ^*Cre*^ *ROSA* ^*RFP*^ mice, suggesting a proliferation or differentiation defect (Supplemental Fig. 1a). In fact, when LN lymphocytes were rechallenged with MOG_35–55_ in the presence or absence of IL-23 following the first immunization with MOG_35–55_, Th17 cell expansion and IL-17A production were greatly diminished in *S1P*_*1*_^*Flox*^ *IL-*1*7A* ^*Cre*^ *ROSA* ^*RFP*^ mice compared with controls (Fig. 1e,f) which may be due to either reduced frequency of Th17 cells or a compromised responsiveness to antigen and IL-23.

### T_reg_-specific deletion of S1P_1_ causes lymphadenopathy and multi-organ inflammation

**Figure 2:**
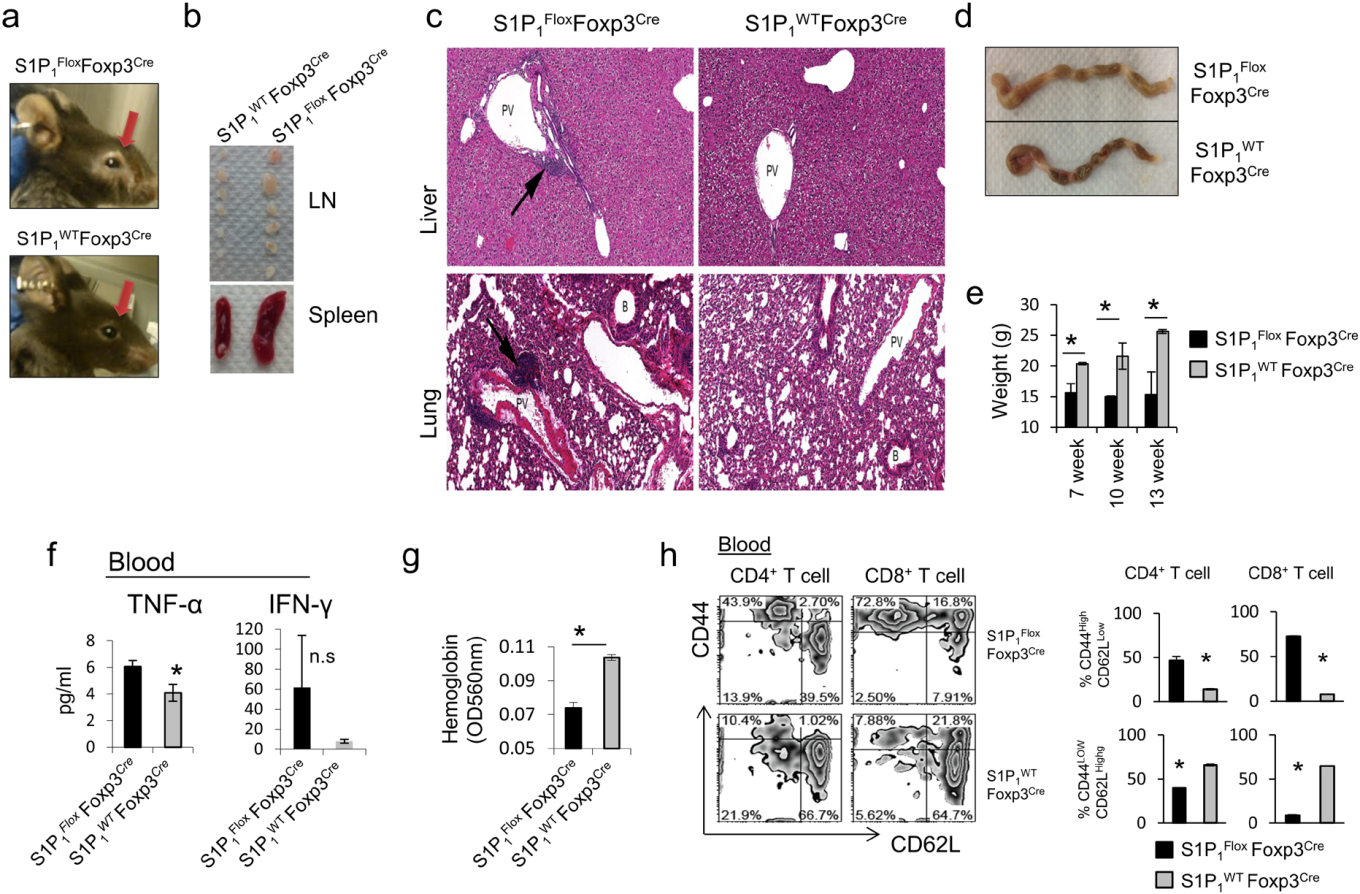
T_reg_-specific deletion of S1P_1_ causes autoimmunity. (**a**) Representative image of skin lesions that develop around the eyes of 8-week-old *S1P*_*1*_^*Flox*^ *Foxp3* ^*Cre*^ compared to *S1P*_*1*_^*WT*^ *Foxp3* ^*Cre*^ mice. (**b**) Representative splenomegaly and lymphadenopathy in 8 weeks-old *S1P*_*1*_^*Flox*^ *Foxp3* ^*Cre*^ mice compared to *S1P*_*1*_^*WT*^ *Foxp3* ^*Cre*^ mice. (**c**) Representative section of liver and lung stained with hematoxylin & eosin (H&E) depicting higher lymphocytic infiltrates in 6 weeks-old *S1P*_*1*_^*Flox*^ *Foxp3* ^*Cre*^ mice. Arrows point the lymphocytic infiltrates. PV, portal vein in liver; PV, pulmonary vein in lung; B, bronchiole. (**d**) Colon inflammation and discoloration in 8-week-old *S1P*_*1*_^*Flox*^ *Foxp3* ^*Cre*^ mice compared to *S1P*_*1*_^*WT*^ *Foxp3* ^*Cre*^ mice. (**e**) Weight chart indicating lack of weight gain over time in *S1P*_*1*_^*Flox*^ *Foxp3* ^*Cre*^ mice. (*) indicates significance, p < 0.05. (**f**) Elevated systemic TNF-α and IFN-γ levels in the serum of 8 weeks-old *S1P*_*1*_^*Flox*^ *Foxp3* ^*Cre*^ mice. (*) indicates significance, p < 0.05. (**g**) Colorimetric assay measuring hemoglobin in adult *S1P*_*1*_^*Flox*^ *Foxp3* ^*Cre*^ mice (*) indicates significance, p < 0.05. A total of 7 to 15 mice per group used for experiments (**e–g)**. (**h**) Activation status of CD4+ or CD8+ T cells in the blood of 8-week-old *S1P*_*1*_^*Flox*^ *Foxp3* ^*Cre*^ compared to *S1P*_*1*_^*WT*^ *Foxp3* ^*Cre*^ mice by CD44 and CD62L staining (left) and quantified (right). 3 mice per group used.

To gain insight into how S1P_1_ regulates T_reg_ biology, we crossed *S1P*_*1*_^*Flox*^ to *Foxp3* ^*Cre*^ mice^4^. *Foxp3* ^*Cre*^ locus harbors an internal ribosomal entry site (IRES)-driven YFP and allows tracking of T_reg_ cells. T_reg_-specific deletion of S1P_1_ has been verified (Supplemental Fig. 1b). *S1P*_*1*_^*Flox*^ *Foxp3* ^*Cre*^ mice started to develop systemic autoimmunity at 8 weeks of age and died at around 13 weeks. We observed skin lesions around the eye with low penetrance (~41%) in these mice (Fig. 2a). At necropsy, we observed enlarged lymph nodes and spleen (Fig. 2b). As the disease progressed, lymphoid organs were observed at necropsy to be smaller than normal. The colon and cecum of 6-week-old *S1P*_*1*_^*Flox*^ *Foxp3* ^*Cre*^ mice was thickened with ill-formed feces suggestive of typhlocolitis. Histologically, the colon showed mild multifocal lymphocytic and neutrophilic colitis with protozoal bloom, mesenteric edema and mild lymphocytic steatitis (data not shown). Lung and liver had mild lymphocytic accumulations in the perivascular and periportal areas, respectively (Fig. 2c,d). Consistent with poor weight gain (Fig. 2e) and multi-organ inflammation, TNF-α and IFN-γ levels were highly elevated in the serum of 8-week-old *S1P*_*1*_^*Flox*^ *Foxp3* ^*Cre*^ mice, which suggests a systemic inflammation (Fig. 2f). Of note, *S1P*_*1*_^*Flox*^ *Foxp3* ^*Cre*^ mice were also anemic (Fig. 2g).

Both CD4^+^ and CD8^+^ T cells in circulation, as well as lymphoid and non-lymphoid organs (Fig. 2h) presented with an activated phenotype. T cells in *S1P*_*1*_^*Flox*^ *Foxp3* ^*Cre*^ mice expressed high levels of CD44 and low levels of CD62L. Additionally, activated CD4^+^ T cells from *S1P*_*1*_^*Flox*^ *Foxp3* ^*Cre*^ mice produced higher quantities of IL-4 and IFN-γ in various organs, including liver, LN and colons compared to their WT counterparts (Supplemental Fig. 1c and data not shown). In the gut lamina propria of *S1P*_*1*_^*Flox*^ *Foxp3* ^*Cre*^ mice, CD4^+^ T cells also produced more IL-17A compared to control *S*1*P*_1_^*WT*^ *Foxp3* ^*Cre*^ mice. Collectively, these results indicated that T_reg_ cells in *S1P*_*1*_^*Flox*^ *Foxp3* ^*Cre*^ mice are unable to suppress adaptive immunity, including Th1, Th2, and Th17 cells, possibly due to defects in mobility and/or functions of T_reg_ cells.

### S1P_1_ regulates T_reg_ cell egress out of lymphoid organs and thus non-lymphoid tissue distribution

**Figure 3:**
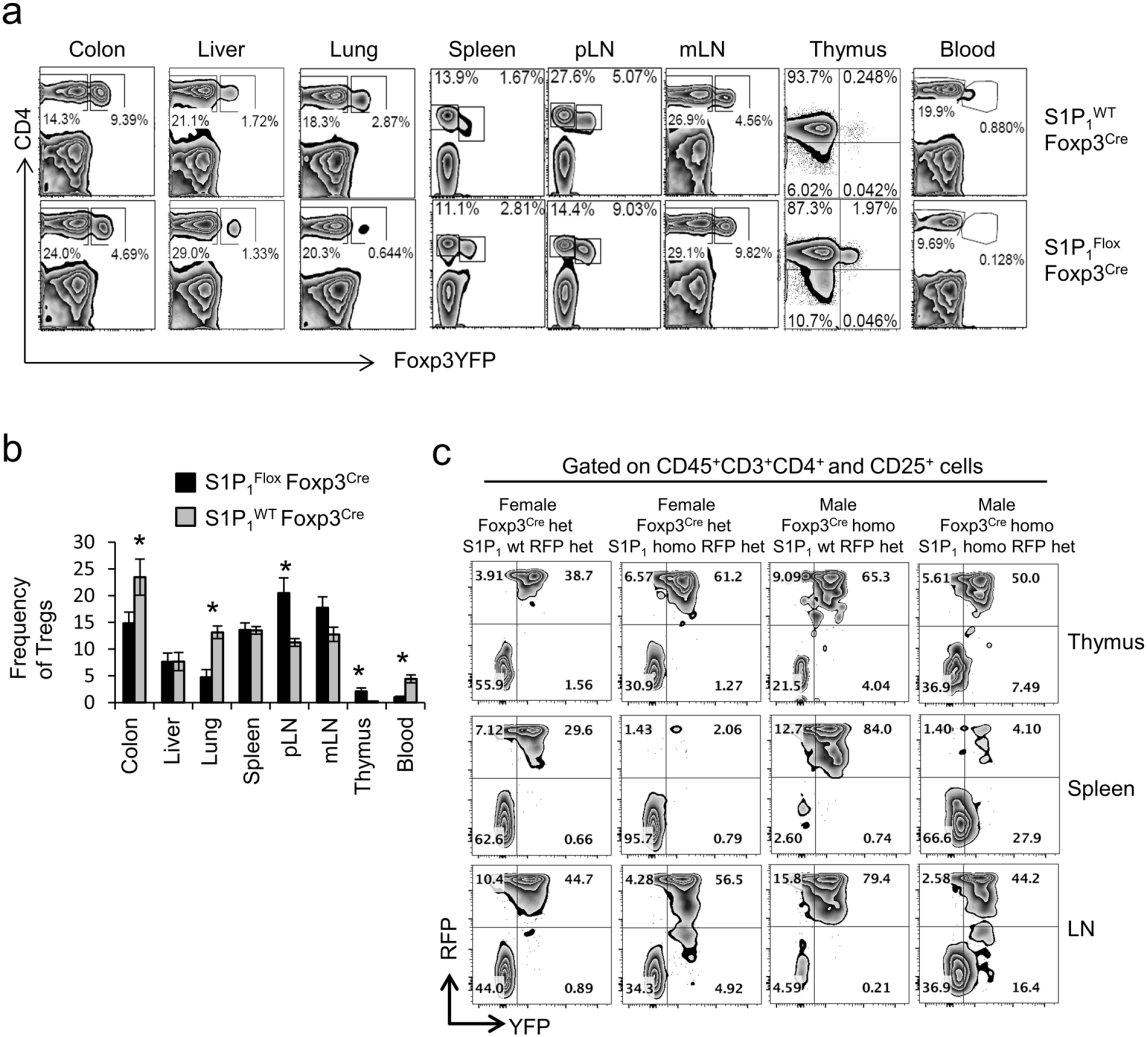
S1P_1_-deficient T_reg_ cells exit thymus but show impaired secondary lymphoid tissue egress and reduced non-lymphoid tissue localization. a) Lymphocytes from various organs of 8-week-old control *S1P*_*1*_^*WT*^ *Foxp3* ^*Cre*^ or *S1P*_*1*_^*Flox*^ *Foxp3* ^*Cre*^ mice were stained for CD4. T_reg_ (CD4^+^Foxp3^+^) distribution was analyzed by flow (representative zebra plots in (**a**) and quantified in (**b**), n = 4–7 mice), (*) indicates p < 0.05 quantified. (**c**) Thymus, spleen and LN from 6–8-week-old mice of indicated genotype were stained for CD45, CD3, CD4, CD25, and CD25^+^CD4^+^CD3^+^ T_reg_ cells gated and among them RFP^+^ or YFP^+^ T_reg_ cells visualized. A representative plot is provided.

S1P_1_ was shown to be critical for the egress of both CD4^+^ and CD8^+^ T cells as well as B cells out of thymus and lymphoid tissues^3,4^. This requirement was demonstrated genetically by conditionally and globally deleting the receptor; and biochemically, using agonists of S1P_1_ receptor. Although the impact of FTY720 treatment on murine T_reg_ cell egress has been assessed^24,25^, the impact of S1P_*1*_ on T_reg_ cells_,_ independently of the effects on other cells, and how S1P_*1*_ regulates specifically T_reg_ cell egress out of thymus and lymphoid organs and homing to various tissues has not been studied. We therefore examined the tissue distribution of T_reg_ cells in adult and neonatal *S1P*_*1*_^*Flox*^ *Foxp3* ^*Cre*^ mice. In adult (8-week-old) mice, we detected increased T_reg_ cell frequency in the thymus (Fig. 3a,b) consistent with previous reports^10,11,22^. This could be due to defective egress out of the thymus or an increased differentiation of T_reg_ cells from CD4^+^CD25^+^ precursors as suggested by others^3,4,22^. We did not however observe a difference in the *in vitro* differentiation efficacy of naïve CD4^+^ T cells into T_reg_ cells in the presence of TGF-β between WT and *S1P*_*1*_^*Flox*^ *Foxp3* ^*Cre*^ (Supplemental Fig. 2b). Similar to the thymus, examination of lymph nodes (brachial, axillary and inguinal) revealed elevated numbers of T_reg_ cells in these organs, which corroborates past studies that suggested a critical role for S1P_1_ in the egress of T_reg_ cells out of lymphoid tissues. In line with this, blood T_reg_ cell levels were greatly diminished in *S1P*_*1*_^*Flox*^ *Foxp3* ^*Cre*^ mice. The fact that the majority of peripheral T_reg_ cells in *S1P*_*1*_^*Flox*^ *Foxp3* ^*Cre*^ mice are Helios^+^ suggests that S1P_1_ is dispensable for the egress of T_reg_ cells out of the thymus, and that compensatory egress receptors may be in place (Supplemental Fig. 2a).

To establish the role of S1P1 in the trafficking behavior of T_reg_ cells under both homeostatic and inflammatory conditions, we performed cell fate mapping experiments. Thus, we crossed *S1P*_*1*_^*Flox*^ *Foxp3* ^*Cre*^ mice with ROSA^RFP^ mice. In these mice T_reg_ cells are labeled with the red fluorescent protein (RFP) once Cre recombinase, which is under the endogenous Foxp3 promoter, has been activated. Thus, in female *Foxp3* ^*Cre*^ *het/S1P*_*1*_^*Flox*^ *homo /ROSA* ^*RFP*^ *het* mice 50% of the T_reg_ cells in lymph nodes is expected to be labeled by both RFP and YFP. The females of this particular mouse strain do not develop inflammation since half of T_reg_ cells behave like WT, whereas males of *Foxp3* ^*Cre*^ */S1P*_*1*_^*Flox*^ *homo/ROSA* ^*RFP*^ develop very severe systemic inflammation. Thus, by studying both males and females of this particular strain we can study the role of S1P_1_ in the trafficking behavior of T_reg_ under both homeostatic and inflammatory conditions. Indeed, we found that almost half of the T_reg_ cells were labeled by both RFP and YFP in LNs but almost-none of these cells were detected in spleens of these animals (Fig. 3c). This result strongly suggests that egress of T_reg_ cells from the thymus is S1P_1_-independent, however S1P_1_-mediated signaling is needed for T_reg_ cells to migrate to the spleen.

We also assessed the non-lymphoid tissue resident T_reg_ cell numbers. Adult *S1P*_*1*_^*Flox*^ *Foxp3* ^*Cre*^ mice had greatly reduced frequency of T_reg_ cells in the colon and lung (Fig. 3a,b). All of the above changes were also readily detectable in 7 to 10-day-old mice, shortly after the initiation of T_reg_ cell development (Supplemental Fig. 2c,d). Additionally, the reduction in T_reg_ cell frequency in liver and spleen was more discernible in young mice than adults. Collectively, these data reveal that S1P_1_ may be dispensable for thymic egress but is required for egress from lymph nodes, and thus possibly indirectly impacts non-lymphoid tissue distribution of T_reg_ cells.

### T_reg_ specific acute deletion of S1P_1_ renders mice more prone to EAE

To study T_reg_-specific S1P_1_ deletion temporally, we crossed *S1P*_*1*_^*Flox*^ mice to *Foxp3* ^*Cre-ERT2*^ mice, which express enhanced green fluorescent protein (eGFP) fused to a Cre recombinase–estrogen-receptor-ligand-binding-domain protein from the 3′ untranslated region of *Foxp3*. This allows deletion of the receptor only after tamoxifen administration. Such acute deletion also provides a time window to study the impact of exclusively S1P_1_ deficiency before the systemic inflammation occurs. Acute deletion of S1P_1_ after five consecutive daily tamoxifen injections resulted in greatly diminished T_reg_ cell percentages and absolute number in circulation, lung and liver. In contrast, lymph node and thymus- resident T_reg_ cells were significantly increased. This was observed only after tamoxifen injection and only in *S1P*_*1*_^*Flox*^ *Foxp3* ^*Cre-ERT2*^ but not control *S*1*P*_1_^*WT*^ *Foxp3* ^*Cre-ERT2*^ mice (Supplemental Fig. 3).

**Figure 4:**
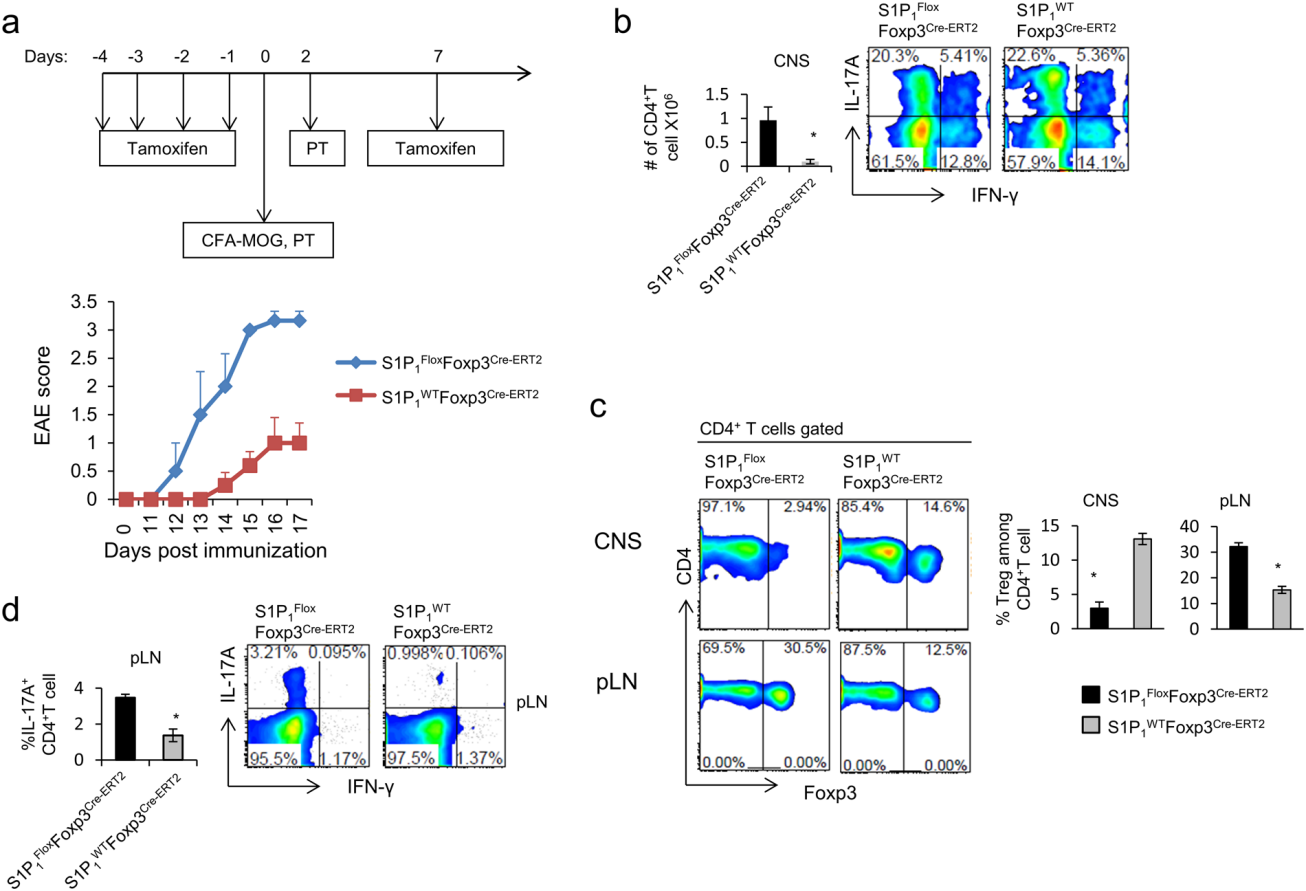
Acute deletion of S1P_1_ in T_reg_ cells renders mice more susceptible to EAE. (**a**) Treatment schematic (Top), and EAE scores after acute deletion of S1P_1_ in *S1P*_*1*_^*Flox*^ *Foxp3* ^*Cre-ERT2*^ and control *S1P*_*1*_^*WT*^ *Foxp3* ^*Cre-ERT2*^ mice_._ (**b**) Absolute number of CNS-infiltrating cells (left), and intracellular staining of CNS-infiltrating CD4^+^ T cells at the peak of disease in test and control groups as treated in (**a**). (**c**) Representative plot for percentage of T_reg_ cell in pLN and CNS at the peak of disease (left) and quantification of T_reg_ percentage (right) in CNS and pLN. (**d**) Th_17_ cells in the pLN at the peak of the disease, intracellular staining of draining pLN lymphocytes 4 hours after stimulation with PMA/Ionomycin.

To explore how S1P_1_ deletion would impact disease onset and progression, we induced experimental autoimmune encephalomyelitis (EAE) by immunization with myelin oligodendrocyte (MOG_35–55_) peptide emulsified in CFA (Complete Freund’s Adjuvant) after acute deletion of S1P_1_ with four consecutive tamoxifen injections. *S1P*_*1*_^*Flox*^ *Foxp3* ^*Cre-ERT2*^ mice developed more severe EAE with earlier disease onset compared to control *S*1*P*_1_^*WT*^ *Foxp3* ^*Cre-ERT2*^ mice, which also received tamoxifen (Fig. 4a). The severity of the disease correlated with lymphocyte infiltration to the brain and spinal cord, thus, higher number of lymphocytic infiltrates and IFN-γ^+^ and IL-17A^+^ single and double producer CD4^+^ T cells were observed in *S1P*_*1*_^*Flox*^ *Foxp3* ^*Cre-ERT2*^ mice at the peak of the disease (Fig. 4b). Similar to the reduction of S1P_1_-deficient T_reg_ cells observed in non-lymphoid tissues at steady state, the ratio of T_reg_ cells among CD4^+^ T cells in the central nervous system (CNS) of *S1P*_*1*_^*Flox*^ *Foxp3* ^*Cre-ERT2*^ mice during EAE was reduced by 4–5-fold (Fig. 4c). While the proportion of S1P_1_-deficient T_reg_ is dramatically reduced in the CNS, their absolute number is slightly higher in comparison to control mice (Supplemental Fig. 3b). These data are in line with our previous finding that the ratio between T_reg_ and the effector T cells is the determining factor that dictate the development of EAE. T_reg_ are unable to suppress EAE if they are outnumbered by effector T cells^26^. Of note, injection site draining lymph nodes of *S1P*_*1*_^*Flox*^ *Foxp3* ^*Cre*^ mice contained 2–3-fold more T_reg_ cells compared to their WT counterparts, which is in accord with the steady state data (Fig. 4c). Interestingly, despite this increase in nodal T_reg_ cellularity, we observed significantly more IL-17A^+^ CD4^+^ T cells in the draining lymph nodes of *S1P*_*1*_^*Flox*^ *Foxp3* ^*Cre-ERT2*^ mice (Fig. 4d). This suggests that S1P_1_-deficient T_reg_ cells may be functionally defective *in vivo*. This defect appears to be more prominent in the containment of Th17, but not Th1 cells in the LN.

### S1P_1_ regulates T_reg_ cell transcriptional program and inhibits central to effector T_reg_ phenotypic switch

**Figure 5:**
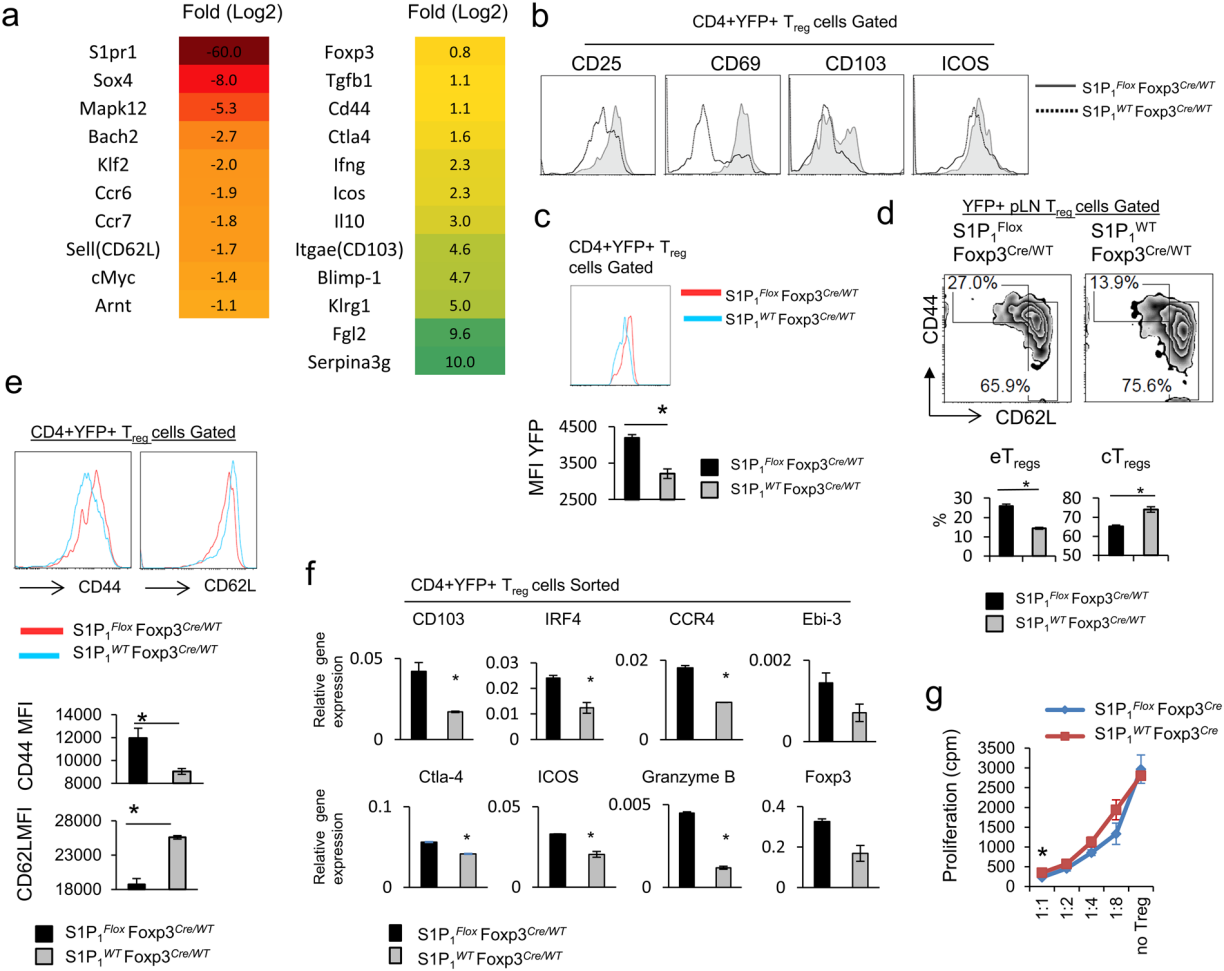
S1P_1_ deletion intrinsically alters T_reg_ transcriptional program and promotes central to effector T_reg_ switch. (**a**) Heat Map of differentially regulated transcripts in T_reg_ cells purified from 8 week-old control *S1P*_*1*_^*WT*^ *Foxp3* ^*Cre*^ and *S1P*_*1*_^*Flox*^ *Foxp3* ^*Cre*^ mice and (**b**). Surface expression of indicated T_reg_ associated markers on YFP^+^ WT (dashed line) or S1P_1_ KO (solid filled line) T_reg_ cells isolated from the LN of healthy female *S1P*_*1*_^*WT*^ *Foxp3* ^*Cre/WT*^ mice as control, or *S1P*_*1*_^*Flox*^ *Foxp3* ^*Cre/WT*^ mice as KO source (harboring 50% WT 50% S1P_1_KO T_reg_ due to random X inactivation), and (**c**) quantification of YFP-Foxp3 protein expression by MFI. d) YFP^+^ WT or YFP^+^S1P_1_KO T_reg_ cells among LN lymphocytes of healthy female *S1P*_*1*_^*WT*^ *Foxp3* ^*Cre/WT*^ or *S1P*_*1*_^*Flox*^ Foxp3^*Cre/WT*^ mice gated. Higher percentages of eT_reg_ cells (CD44^+^CD62L^low^) are observed (bar graphs in (**d**) bottom panel) and MFI values of CD44 and CD62L are charted for quantification purposes in (**e**). (**f**) YFP/Foxp3^+^ T_reg_ cells were sorted from control *S1P*_1_^*WT*^ *Foxp3* ^*Cre/WT*^ or *S1P*_*1*_^*Flox*^ *Foxp3* ^*Cre/WT*^ female mice and RNA was extracted. Relative gene expression of indicated eT_reg_ associated genes was quantified by real time qPCR. Higher levels of indicated genes detected in S1P_1_KO T_reg_ cells. (**g**) *In vitro* suppression assay performed with T_reg_ cells sorted from 8-week-old WT or *S1P*_*1*_^*Flox*^ *Foxp3* ^*Cre*^ mice. (*) indicates <0.05. n = 3–5 mice per group.

To examine the functional and phenotypic impact of S1P_1_ on T_reg_ cell biology we sorted CD4^+^YFP^+^ T_reg_ cells from 8-week-old WT and *S1P*_*1*_^*Flox*^ *Foxp3* ^*Cre*^ mice, and analyzed the global transcriptional changes via RNA-sequencing. A select list of genes is presented in the heat map in Fig. 5a. The selected genes were validated via real-time qPCR (Supplemental Fig. 4a). Along with previously defined upregulation of CD69, CD103, CTLA-4, ICOS, CD25, we realized that transcriptional changes suggested enrichment of the effector T_reg_ cell subset in *S1P*_*1*_^*Flox*^ *Foxp3* ^*Cre*^ mice: the levels of CCR7 and CD62L were strongly downregulated, CD44, KLRG1, CD69, CD103, CTLA-4 and ICOS mRNA levels were upregulated. Consistent with the mRNA data, CD44^high^CD62L^low^ eT_reg_ cells were more predominant than CD44^low^CD62L^high^ cT_reg_ cells and the surface expression of KLRG1, CD69, CD103, CTLA-4, ICOS molecules were elevated in T_reg_ cells obtained from *S1P*_*1*_^*Flox*^ *Foxp3* ^*Cre*^ mice (Supplemental Fig. 4b–d). In addition, both mRNA and protein levels of Foxp3 were increased in S1P_1_-deficient T_reg_ cells. (Not shown) As previously demonstrated to be a characteristic of eT_reg_ cells, we observed higher rates of apoptosis in T_reg_ cells purified from *S1P*_*1*_^*Flox*^ *Foxp3* ^*Cre*^ mice (Supplemental Fig. 4e). Since *S1P*_*1*_^*Flox*^ *Foxp3* ^*Cre*^ mice have autoimmunity, it is possible that the increased number of effector T_reg_ cells that we observed in the *S1P*_*1*_^*Flox*^ *Foxp3* ^*Cre*^ mice could be the result of systemic inflammation and does not necessary imply that S1P_1_ regulates the transition from cT_reg_ to eT_reg_. Thus, we examined the S1P_1_KO T_reg_ phenotype in two non-inflammatory settings. To this end, we used *S1P*_*1*_^*Flox*^ *Foxp3* ^*Cre/WT*^ healthy female mice which have 50% WT 50% S1P_1_ KO T_reg_ cells due to random X-inactivation, owing to *Foxp3* being an X-linked gene. YFP^+^ T_reg_ cells sorted from the LN of these non-inflamed mice were phenotypically similar to those obtained from *S1P*_*1*_^*Flox*^ *Foxp3* ^*Cre*^ mice with inflammation with regard to mRNA and protein expression (Fig. 5b–f). More specifically, we detected higher cell surface expression of KLRG1, CD69, CD103, CTLA-4, ICOS and elevated frequency of CD44^high^CD62L^low^ eT_reg_ cells and reduced frequency of CD44^low^CD62L^high^ cT_reg_ cells. These changes were also reflected in the MFI surface expression of CD44 and CD62L (Fig. 5b–f). Similarly, Foxp3 mRNA and protein levels were upregulated in S1P_1_KO T_reg_ cells compared with WT T_reg_ (Fig. 5c). We obtained identical results with mixed bone marrow chimeric mice (not shown). These data suggest that S1P_1_ inhibits central to effector T_reg_ cell conversion.

Moreover, to further dissociate the impact of inflammation from S1P_1_ signaling itself we examined T_reg_ cells in our acute S1P_1_ deletion model. Post-tamoxifen treatment, S1P_1_-deficient splenic T_reg_ cells had an effector memory phenotype as shown by the expression of higher levels of CD44, CD103 and CD69 and low CD62L (Supplemental Fig. 5a). In accord with the protein data, sorted T_reg_ cells from lymph nodes and spleen of *S1P*_*1*_^*Flox*^ *Foxp3* ^*Cre-ERT2*^ mice post-tamoxifen treatment had elevated expression of various effector memory T_reg_-associated genes, including *cd*1*03*, *blimp-1*, *Ccr4* and *ifr4* (Supplemental Fig. 5b). We also detected higher Foxp3 mRNA in these mice. Consistent with the previous reports suggesting an apoptosis prone nature for effector T_reg_ cells^16,18^, splenic T_reg_ cells from *S1P*_*1*_^*Flox*^ *Foxp3* ^*Cre-ERT2*^ mice post-tamoxifen treatment were significantly more annexin V^+^ (Supplemental Fig. 5c). Altogether, these results are in line with our observations in the chronic deletion and chimeric mice, and thus, support the notion that S1P_1_ regulates the egress of T_reg_ cells as well as their phenotypic diversity.

Lastly, to test the functionality of S1P_1_KO T_reg_ cells, we performed an *in vitro* suppression assay. S1P_1_-deficient and WT T_reg_ cells were both capable of inhibiting the proliferation of effector T cells (Fig. 5g) Of note, we also did not detect a difference in IL-2 responsiveness of S1P_1_KO and WT T_reg_ cells, as measured by STAT5 phosphorylation and dose-dependent proliferation in response to IL-2 (Supplemental Fig. 5d,e). Collectively, these results suggest that S1P_1_ expression on T_reg_ cells is crucial for their *in vivo* suppression function by controlling their trafficking behavior and their transition from cT_reg_ to eT_reg_.

### Effector T_reg_ cells are enriched in fingolimod-receiving MS patients

**Figure 6:**
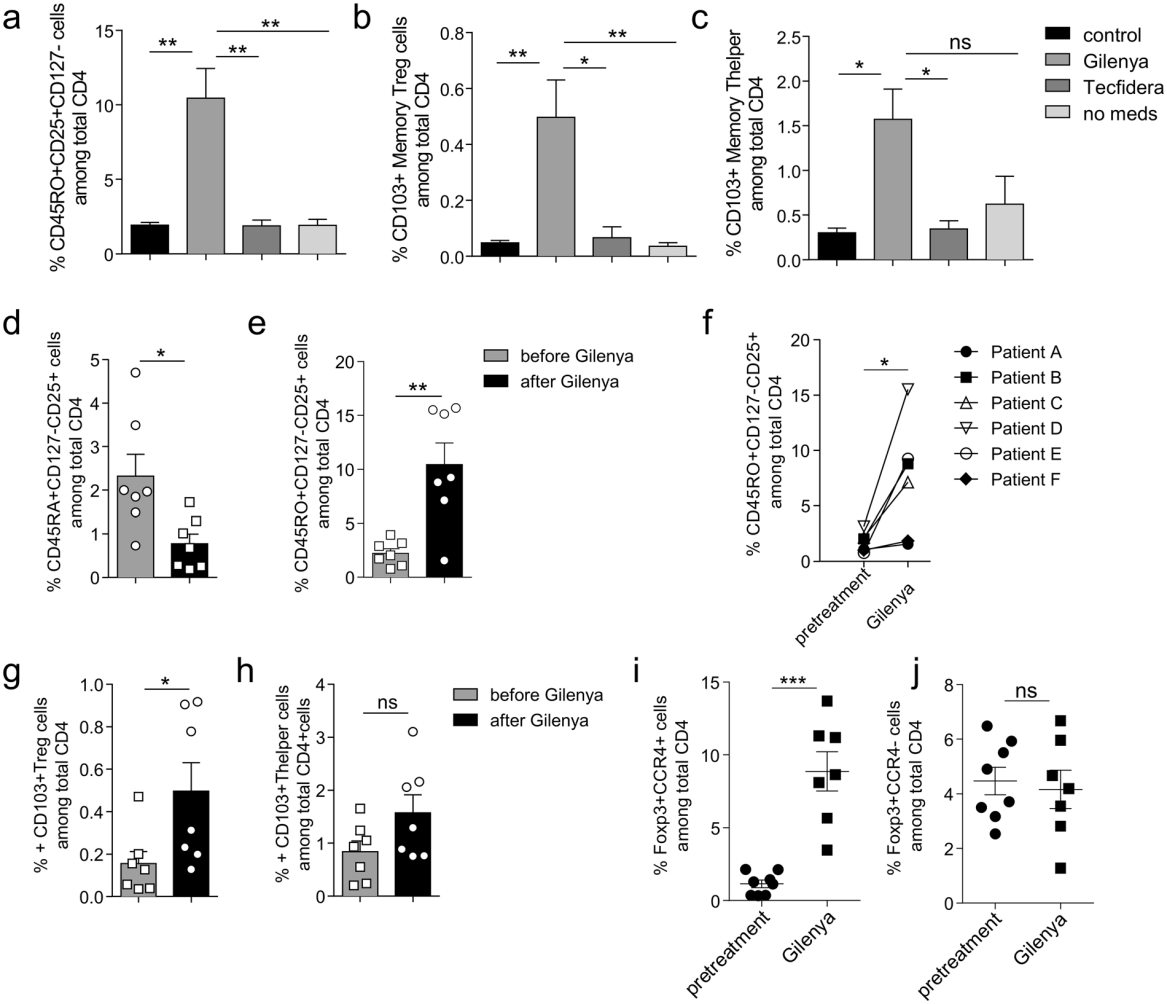
Fingolimod promotes the conversion of cT_reg_ cells to eT_reg_ cells in MS patients. (**a**) Percentage of CD4^+^CD45RO^+^CD25^+^CD127^−^ memory T_reg_ cells in healthy controls, untreated MS patients (*no meds*), MS patients treated with fingolimod or dimethyl fumarate. Percentage of CD103+ T_reg_ cells (CD4^+^CD45RO^+^CD25^+^CD127^−^) (**b**) and CD103^+^ T helper cells (CD4^+^CD45RO^+^CD25^−^CD127^+^) (**c**) in the same cohort of MS patients and healthy controls as in (**a**). Data are mean ± SEM of five or six donors in each group. *p < 0.05; **p < 0.01; ***p < 0.001 (ANOVA). Comparison of the number of naïve (CD45RO^−^) (**d**) or memory (**e**) T_reg_ cells (CD45RO^+^) in MS patients before (gray bars) and after (black bars) fingolimod treatment. (**f**) Increase in Memory T_reg_ cells in individual MS patients after fingolimod treatment. CD103 expression on T_reg_ (**g**) and T helper (**h**) cells from MS patients before (grey bars) and after (black bars) fingolimod treatment. Percentage of Foxp3^+^CCR4^+^ (**i**) and Foxp3^+^CCR4^−^ (**j**) T cell subsets in MS patients before (filled circle) and after (filled square) fingolimod treatment. Data are mean ± SEM of five to seven donors. *p < 0.05; **p < 0.01; ***p < 0.001 (two-tailed paired *t* test). All data are presented as frequency among total CD4^+^ T cells.

To assess if the effects of S1P_1_ signaling observed in mice also occur in human T_reg_ cells, we compared blood samples obtained from relapsing remitting MS (RRMS) patients treated with the S1P_1_ inhibitor, fingolimod, to subjects with MS treated with dimethyl fumarate treatment, or no therapy, with age and gender matched healthy controls (Fig. 6). The patients treated with fingolimod showed significant lymphopenia in their blood, as expected (not shown). More importantly, T_reg_ frequency with respect to total CD4^+^ T cells was no different between the patient cohorts, (Supplemental Fig. 6a) but the fingolimod-treated subjects had a significantly elevated percentage of CD4^+^CD45RO^+^CD25^high^CD127^low^ memory T_reg_ cells in the blood compared to patients under dimethyl fumarate, no treatment or healthy controls (Fig. 6a and Supplemental Fig. 6a). A small fraction of human memory T_reg_ cells in peripheral blood, similar to murine eT_reg_ cells, also express CD103. The frequency of CD103^+^ memory T cells was significantly higher in fingolimod-treated MS patients in both T_reg_ cells (Fig. 6b) and their T helper (T_H)_ cell counterpart (Fig. 6c). Comparison of PBMCs from the same patients before and after fingolimod treatment demonstrates that fingolimod promotes the conversion of naïve/central T_reg_ cells to a memory/effector phenotype over time (Fig. 6d–f and Supplemental Fig. 6b). More specifically we analyzed CD103^+^ T_reg_ cells before and after treatment, which followed a similar pattern (Fig. 6g and h). Additionally, when we evaluated T_reg_ cells by expression of Foxp3 and CCR4, we found that the subset of Foxp3^+^CCR4^+^ T_reg_ cells is greatly expanded after fingolimod treatment, with Foxp3^+^CCR4^−^ cells remaining the same (Fig. 6i and j). It is noteworthy that CD4^+^CD25^−^CD127^high^ T_h_ cells also exhibit enhanced CD103 expression, suggesting that S1P_1_ signaling may maintain a central memory/naïve program in conventional T cells as well (Fig. 6c and h). Thus, these observations are in line with the mouse data presented above and support our observations that S1P_1_ signaling inhibits the switch from a central to an effector memory T_reg_ cell phenotype. Therefore, in the absence of S1P_1_ signaling, either through genetic deletion or down-modulation of cell surface expression by biochemical intervention through an antagonist (fingolimod), phenotypic conversion of cT_reg_ to eT_reg_ occurs.

## Discussion

The role of S1P_1_ in regulating Th17 biology has not been investigated previously. Genetic deletion of S1P_1_ from Th17 cells in this study revealed novel information in this regard. We show that this receptor is necessary for Th17 cell homing to peripheral organs, CNS and for EAE pathogenesis. Additionally, our data suggest that Th17 generation may be regulated by S1P_1_ as indicated by reduced Th17 levels in *S1P*_*1*_^*Flox*^ *IL-17A* ^*Cre*^ mice following immunization with MOG_35–55_. Involvement of S1P_1_ in Th17 development has been suggested by Garriss *et al*. recently and is supported by our observations.

Previous work by Matloubian *et al*. and Allende *et al*. demonstrated a requirement for S1P_1_ receptor for the egress of T cells out of thymus and lymph nodes. Although these studies suggested that T_reg_ cells would also rely on S1P_1_, direct and T_reg_-specific assessment of its functions was lacking. More recently, Liu *et al*. investigated this elegantly by using CD4^cre^ deletion of S1P_1_ which alters receptor levels in conventional CD4^+^ T cells and thus T_reg_ precursors as well. They utilized CD2 promoter driven S1P_1_ transgenic mice, which strengthened their findings. Their observations revealed an unprecedented inhibitory role for S1P_1_ during differentiation of T_reg_ cells from CD4^+^CD25^+^Foxp3^−^ precursors. In other words, CD4-specific deletion of S1P_1_ resulted in more and functionally better T_reg_ cells whereas CD2-driven overexpression of S1P_1_ yielded fewer T_reg_ cells with poor suppressive functions. However, several points remained elusive in these previous works. Does S1P_1_ regulate the functions of committed T_reg_ cells, that is, after Foxp3 is turned on? Does altering S1P_1_ level exclusively in T_reg_ cells result in the same phenotype as mutants that target the receptor broadly, including conventional T cells? Is the lymphoid and non-lymphoid tissue distribution of T_reg_ cells altered by specific S1P_1_ ablation? *S1P*_*1*_^*Flox*^ *Foxp3* ^*Cre*^mice proved useful for addressing these questions. Despite the reports suggesting that S1P_1_-deficient T_reg_ cells are better suppressors, *S1P*_*1*_^*Flox*^ *Foxp3* ^*Cre *^mice had an autoimmune phenotype resembling that of *scurfy* mice with some delay in development of fulminant autoimmunity. Although both CD4^+^ and CD8^+^ T cells, as well as B cells, were activated, CD8^+^ T cells showed massive expansion and were the major source of elevated systemic TNF-α and IFN-γ in these mice. This phenotype was suggestive of some *in vivo* functional impairment unique to T_reg_ cells.

Because lymphocyte egress is the primarily studied cellular response regulated by S1P_1_, close examination of T_reg_ cell tissue distribution across lymphoid and non-lymphoid organs in *S1P*_*1*_^*Flox*^ *Foxp3* ^*Cre*^ mice revealed major defects. Consistent with the previous work investigating conventional T cell egress^3,4^, and T_reg_ cell egress using CD4^cre^ system^22^, Foxp3^Cre^-specific S1P_1_ deletion resulted in higher frequency of T_reg_ cells in thymus and LNs. Liu *et al*.’s work using CD4^Cre^ system *in vitro* showed that T_reg_ cell differentiation from S1P_1_-deficient CD4^+^ naïve T cells is more efficient. However, in this system, S1P_1_ was deleted in all T cells prior their commitment to either conventional or regulatory T cells. The observation in our study that *in vitro* differentiation of naïve CD4^+^ T cells obtained from *S1P*_*1*_^*Flox*^ *Foxp3* ^*Cre*^ mice is equivalent to that of WT indicates that S1P_1_ deletion after commitment to the T_reg_ cell lineage does not impact their differentiation efficacy at least *in vitro* in the presence of TGF-β. However, *in vivo*, the evidence Liu *et al*. presents regarding refractory role of S1P_1_ in T_reg_ development is also supported by our data. T_reg_ cells are found in increased numbers in LNs and comparable numbers in the spleens of *S1P*_*1*_^*Flox*^ *Foxp3* ^*Cre*^ adult mice while there is a buildup of T_reg_ cells in thymus in those mice. Moreover, *Foxp3* ^*Cre*^ *het/S1P*_*1*_^*Flox*^ *homo/ROSA* ^*RFP*^ *het* mice also show S1P_1_ KO T_reg_ homing to LNs, therefore S1P_1_ may not be an absolute requirement for T_reg_ cell egress out of the thymus, as it is conceivable that T_reg_ cells may be utilizing receptor/s other than S1P_1_ to egress thymus for blood. T_reg_ cells also express the remaining four S1P receptors with S1P_4_ levels being comparable to S1P_1_. One or more of the receptors may play a role in this process and explain the leaky egress in the absence of S1P_1_^25^. Further study is needed to verify this. Our data, supporting previous studies, also show that egress of T_reg_ cells out of lymph nodes into lymph requires S1P_1_, as evidenced by increased T_reg_ cells in LNs. Due to this defective lymphoid tissue egress, we hypothesize that non-lymphoid tissue homing of T_reg_ cells are adversely affected. Indeed, our examination of various non-lymphoid organs such as lung, liver and colon showed reduced T_reg_ cellularity in both *S1P*_*1*_^*Flox*^ *Foxp3* ^*Cre*^ mice and after acute deletion of S1P_1_ in the tamoxifen inducible system. Given the critical role of tissue resident T_reg_ cells in preventing autoreactive T cells at these sites, multi-organ inflammation in *S1P*_*1*_^*Flox*^ *Foxp3* ^*Cre*^ mice could partly be explained by reduced T_reg_ cellularity in the peripheral organs. These observations also underline the requirement for the presence of T_reg_ cells in the tissues to suppress autoimmunity rather than lymph nodes. This finding provides some insight into repercussions of long term fingolimod use in MS patients. Our results suggest that patients under fingolimod treatment are expected to have a reduction in tissue resident T_reg_ cells, since fingolimod also causes downregulation of S1P_1_. Although the number of Foxp3^−^ conventional T cells would follow a similar decrease in response to fingolimod, a more precise kinetic analysis of T_reg_ vs non-T_reg_ T cell disappearance from tissues is needed to evaluate the impact of T_reg_ loss on activation of autoreactive tissue-resident T cells. Nevertheless, such reduction in tissue-resident T_reg_ numbers may result in loss of tolerogenic properties of antigen presenting cells which may become crucial in driving autoreactive T cells in some microenvironments and individuals once lymphopenia is resolved due to cessation of treatment.

Another major finding of this report is that S1P_1_ signaling plays a critical role in maintaining central memory phenotype of T_reg_ cells. This was demonstrated by comparison of the gene expression profile, as well as cell surface marker expression of T_reg_ cells obtained from *S1P*_*1*_^*Flox*^ *Foxp3* ^*Cre*^ mice to that of controls. Similarly, S1P_1_-deficient T_reg_ cells obtained from female *S1P*_*1*_^*Flox*^ *Foxp3* ^*Cre/WT*^ or bone marrow chimeras or, *S1P*_*1*_^*Flox*^ *Foxp3* ^*Cre-ERT2*^ mice after acute receptor deletion assumed effector phenotype indicating that the enrichment of effector T_reg_ cells is due to the absence of S1P_1_ signaling, but not due to inflammatory signals. More importantly, this phenomenon could be recapitulated in MS patients treated with S1P_1_ antagonist fingolimod, but not other drugs such as Tecfidera, nor in healthy controls. How S1P_1_ regulates the cT_reg_ to eT_reg_ conversion is not clear. We could not implicate blimp-1 in this process because it was upregulated in the absence of S1P_1_ in the inflammatory setting but not in non-inflammatory setting. It was previously shown that *irf4* is required for conversion of cT_reg_ into eT_reg_. We also observed an increase in *irf4* transcript levels in T_reg_ cells obtained from *S1P*_*1*_^*Flox*^ *Foxp3* ^*Cre-ERT2*^ mice after acute receptor deletion. However, it is unknown if overexpression of *irf4* is sufficient for this conversion, as is how S1P_1_ signaling restrains *irf4* expression.

Compelling evidence from past *in vitro* and *in vivo* studies indicates that FTY720 (fingolimod)-conditioned T_reg_ cells become more suppressive with regard to suppression of proliferation of target CD4^+^ T cells, or inhibition of IL-4 and IFN-γ cytokine production by target cells^24,25^. Additionally, they suppress airway inflammation in a mouse model more efficiently^25^. More recent reports showed enhanced *in vitro* suppressive function of S1P_1_KO T_reg_ cells. Conversely, reduced suppressive capacity of S1P_1_ overexpressing transgenic T_reg_ cells has been reported^22,23^. However, T_reg_ cell suppression of target cells can be achieved by various means, and not all aspects of suppression have been tested in those studies. Despite the fact that eT_reg_ cells appear to express components of the molecular machinery utilized for target cell suppression at high levels (ICOS, CTLA4, CD25, Foxp3) and appear to be potent suppressors *in vitro*, the question still remains as to what would happen if the balance between eT_reg_ vs cT_reg_ were to shift in favor of the former in an organism *in vivo*. The acute deletion model was proved to be more informative in this regard. Our data from the EAE model showed higher IL-17A producing CD4^+^T cells in the draining lymph nodes of MOG_35–55_ peptide immunized *S1P*_*1*_^*Flox*^ *Foxp3* ^*Cre-ERT2*^ mice after acute receptor deletion, despite the presence of 3-fold higher numbers of eT_reg_ cells compared with WT. We hypothesize that this may likely be as a result of improper access of eT_reg_ cells to T cell zones due to their reduced expression of CCR7, which is required for this process^19^. Thus, in *S1P*_*1*_^*Flox*^ Foxp3^*Cre*^ mice, in addition to the reduced non-lymphoid organ access of T_reg_ cells, T_reg_ cells that are trapped in the secondary lymphoid organs will have problems accessing the T cell zones, and will therefore be unable to control autoreactive T cells that were primed with migrant DCs carrying autoantigens from tissues that are devoid of T_reg_ cells. Alternatively, S1P_1_KO T_reg_ cells may also be functionally incapable in certain aspects of their suppression machinery. This hypothesis has merit, given that Garris *et al*. have recently established that STAT3 is activated by S1P_1_ signaling^27^. A more recent study showed FTY720 can downmodulate STAT3 phosphorylation in a murine model which has overactive STAT3 due to an internalization-defective S1P_1_^28^. Prior to this finding, it was shown that STAT3-deficient T_reg_ cells were unable to perform suppression of exclusively Th17 cells *in vivo*, despite their intact *in vitro* suppression ability^29,30^. Our observation of higher IL-17 responses in the draining lymph nodes of *S1P*_*1*_^*Flox*^ *Foxp3* ^*Cre-ERT2*^ mice after acute receptor deletion and immunization corroborates this notion of a customized T_reg_ cell subset for the suppression of Th17 cells. However, in our hands, addition of S1P_1_ agonists did not impact IL-6 or IL-23 driven STAT3 phosphorylation. We utilized phospho-flow technique in our studies. Additionally, the previous papers tested this phenomenon on cells which have already higher pSTAT3 levels. These differences might account for the variation between our and previous results.

T_reg_ cells are critical for immunologic tolerance, and containment of protective immune responses. As such, they are of critical importance for the suppression of myelin specific autoreactivity during multiple sclerosis. A growing number of reports revealed unexpected exacerbation in MS features of patients who are switching to fingolimod or of those who stopped its use, suggesting an unknown immunomodulation. In this study, we attempted to explore fingolimod’s potential effects on T_reg_ cells through S1P_1_ using T_reg_-specific genetic deletion of the receptor. In summation, our results show that S1P_1_ is critical for the lymphoid tissue exit of T_reg_ cells, and subsequent localization to non-lymphoid tissues. Thus, disruption of S1P_1_ signaling permanently in T_reg_ cells causes autoimmunity in mice, whereas a temporal disruption renders mice more susceptible to the development of EAE, the animal model of multiple sclerosis. More importantly, our results reveal that, apart from egress, S1P_1_ regulates various aspects of cT_reg_ to eT_reg_ phenotypic conversion; as such, loss of S1P_1_ signaling promotes conversion to effector phenotype in both mice and humans, which might have repercussions for the functions of T_reg_ cells. These results provide novel insight in to the possible effects of long-term fingolimod use in MS patients on T_reg_ cells.

## Methods

### Human Samples

Frozen PBMCs were obtained from participants in the Benaroya Research Institute (BRI) Immune Mediated Disease (IMD) Registry; informed consent was obtained after the nature and possible consequences of the studies were explained. Three patient cohorts (based on disease modifying therapy treatment: Gilenya (fingolimod), Tecfidera (dimethyl fumarate) and no treatment) and healthy controls were selected for these studies. In addition, frozen PBMCs from the same MS patients prior and under Gilenya treatment were analyzed. The diagnosis of RRMS was based on Revised McDonald Diagnostic Criteria for MS (C. H. Polmanetal., Diagnostic criteria for multiple sclerosis: 2005 revisions to the “McDonald Criteria”)^31^. All subjects in the MS patient/no treatment cohort were off immunomodulating and immunosuppressive therapies at the time of study and for at least 3 months before the blood draw. Control subjects were recruited from the BRI IMD Registry and were selected because of a lack of autoimmune disease or any family history of autoimmunity. The research protocols were approved by the Institutional Review Board at BRI. All methods for human studies involving human samples were performed in accordance with the relevant guidelines and regulations.

### Mice

*S1P*_*1*_^*Flox*^ mouse was a gift from Dr. Richard L. Proia and bred in house to *Foxp3* ^*CreYFP*^, which was a gift from Dr. A. Rudensky, at specific pathogen free conditions. Foxp3^Cre-ERT2-YFP^ mice were purchased from Jackson Laboratories (STOCK Foxp3tm9(EGFP/cre/ERT2) Ayr/J) and bred to *S1P*_*1*_^*Flox*^ and then for some experiments to *ROSA* ^RFP^ on C57BL/6 background. All the mice were bred and maintained under specific pathogen-free conditions. The experiments were approved by the Institutional Animal Care and Use Committee of Seattle Children’s Research Institute. All methods for mice studies involving mouse samples were performed in accordance with the relevant guidelines and regulations.

### EAE induction

EAE was induced by s.c. immunization of mice at the flanks with an emulsion of MOG_35–55_ peptide (100 μg) emulsified in CFA supplemented with 4 mg/ml *Mycobacterium tuberculosis* extract H37Ra (Difco). Additionally, the animals received 200 ng pertussis toxin (List Biological Laboratories) i.p. on days 0 and 2. Clinical signs of EAE were assessed daily and scored according to the following criteria: 0, no signs of disease; 1, limp tail; 2, hind limb weakness; 3, hind limb paralysis; 4, hind limb and forelimb paralysis.

### Tamoxifen treatments

Tamoxifen (Sigma, T5648) was dissolved in corn oil (Sigma, C8267) to a stock solution of 15 mg/ml by overnight shaking at 37 °C and kept at 4 °C until use. Mice were injected intraperitoneally with 5ul/g of stock tamoxifen solution (final 0.75 mg tamoxifen/g mice). Injections were repeated for 5 consecutive days and mice were euthanized on day 6 or 7. For EAE experiments, mice received 4 injections of tamoxifen, and were then immunized, followed by another injection day 7 after EAE induction. For some experiments, 2 consecutive injections were performed and mice were euthanized on day 2 or 3.

### Autoantibody measurement

Anti-dsDNA ELISA on blood serum was performed as described previously^32^. Total serum levels of IgM, IgG, IgG1, IgG2b, and IgA were determined with the ELISA kit from Southern Biotech.

### *In vitro* suppression assay

The assay was performed as described by Chaudhry *et al*.^30^.

### *In vitro* differentiation into Treg cells

CD62L^high^ CD44^low^ naïve CD4^+^ T cells were sorted from WT or *S1P*_*1*_^*Flox*^ *Foxp3* ^*Cre*^ mice (LN and spleen combined) after initial positive enrichment step with CD4^+^ microbeads (Miltenyi Biotech). 1 × 10^5^ T cells were cultured in 24-well plates with 5 × 10^5^ irradiated antigen presenting cells (CD4^−^ fraction) in the presence of 5ng/ml TGF-β and 2 µg/ml anti-CD3 in RPMI supplemented with 10% FBS. 5 days later Foxp3^+^ cells were quantified by YFP or Foxp3 staining.

### Recall responses

Splenocytes or inguinal lymphocytes were prepared from indicated mice at the 7^th^ day of immunization (with Complete Freund’s Adjuvant and MOG_35–55_ emulsion) and restimulated with 100 µg MOG_35–55_ plus recombinant IL-23 or without IL-23 (1 × 10^5^ cells/well) in 96-well plate. Five days later RFP^+^ cells were visualized by Flow cytometry. Cells were stimulated 4 h with PMA/Ionomycin/Golgi Plug to perform intracellular staining for IL-17A and IFN-γ.

### T_reg_ cell proliferation and pSTAT5 detection

T_reg_ cells were sorted from 6 week-old WT or *S1P*_*1*_^*Flox*^ *Foxp3* ^*Cre*^ mice (LN and spleen combined) after initial positive enrichment step via CD4^+^ microbeads (Miltenyi Biotech). Treg cells (2.5 × 10^4^) were co-cultured with 1.25 × 10^5^ irradiated antigen presenting cells (CD4^−^ fraction) and 1 µg/ml anti-CD3 plus increasing concentrations of IL-2 (0, 1, 100, 1000 Units) in 96-well round bottom plates in RPMI supplemented with 10% FBS. 16 h prior to harvesting, cells were pulsed with 1µCi^3^H-Thymidine. At 72 h, cells were harvested and thymidine incorporation was measured with a scintillation counter.

To examine IL-2 signaling, 1 × 10^6^ lymphocytes from LN of WT or *S1P*_*1*_^*Flox*^ *Foxp3* ^*Cre*^ mice or female *S1P*_*1*_^*Flox*^ *Foxp3* ^*Cre/WT*^ mice were stimulated with varying concentrations of IL-2 for 20 minutes at 37 °C in 96-well plate (100 µl volume). Cells were fixed with the addition of equal volume of 4% Formalin for 10 minutes. Cells were permeabilized with Perm Buffer III (BD Biosciences) and staining of pSTAT5 was performed per manufacturer’s protocol.

### Generation of bone marrow chimeras

The recipient *Rag* ^*−/−*^ mice were irradiated 4–6 h before transfer at 550 rad and treated with antibiotics (Baytril) for 2 weeks. Irradiated mice received retro-orbitally 50/50 mixture of CD45.1^+^WT and CD45.2 *S1P*_*1*_^*Flox*^ *Foxp3* ^*CreYFP*^ mice bone marrow cells (5 × 10^6^) which are depleted of T cells by CD90.2 microbeads (Miltenyi Biotech). The mice were analyzed 5–8 weeks post-transfer.

### RNA sequencing

Total RNA was purified from T_reg_ cells sorted via FACSAria gating on Foxp3YFP^+^ CD4^+^ cells. Total RNA was purified using the RNeasy mini kit (Qiagen). RNA sequencing was performed at the Genomic Core Facility Southwestern Medical Center, University of Texas as per the facility protocols.

### Flow cytometry

For flow cytometry, cells from spleen and LNs were isolated and surface stained with the appropriate antibodies. For intracellular cytokine staining, single-cell suspensions were cultured directly in RPMI containing 10% fetal bovine serum (FBS) and Golgi plug for 4–5 h or re-stimulated in RPMI containing 10% FBS with 50ng/ml of phorbol 12-myristate 13-acetate and 1 μg/ml of ionomycin in the presence of Golgi plug. Cells were then fixed and stained according to the instructions from the manufacturer, using an intracellular cytokine staining kit (BD Biosciences). Human PBMC’s were cultured in RPMI 1640 medium supplemented with 2 mM glutamine, 1% (v/v) nonessential amino acids, 1% (v/v) sodium pyruvate, penicillin (50 U/ml), streptomycin (50 μg/ml) (all from Invitrogen), and 5% heat-inactivated human serum. Cells were stained on the surface with the appropriate antibodies. When indicated, cells were then fixed with Fix and Perm buffer (eBioscience), according to the manufacturer’s instructions and stained for Foxp3.

Following antibodies were used for staining: Mice:CD4 (GK1.5, eBioscience), CD44 (IM7, BioLegend), CD62L (MEL-14, eBioscience), CD69 (H1.2F3, eBioscience), CD25 (PC61.5, eBioscience), CD103 (2-E-7, eBioscience), KLRG1 (2F1/KLGR1, BioLegend), ICOS (C398.4 A, BioLegend), CTLA4 (UC10–4B9, eBioscience), CD8 (53–6.7, eBioscience), GranzymeB (NGZB, eBioscience), TNF-α (MP6-XT22, eBioscience), IFN-γ (XMG1.2, BioLegend), IL-17A (eBio17B7, eBioscience), IL-4 (11B11, eBioscience), Foxp3 (MF-14, BioLegend), CD86 (GL1, eBioscience), CD38 (Clone 90, eBioscience), MHCII (M5/114.15.2, eBioscience), CD42d (1C2, eBioscience), Ter119 (TER-119, BioLegend), CD45.1 (A20, eBioscience), CD45.2 (104, eBioscience), Helios (22F6, BioLegend), pSTAT5 (SRBCZX, eBioscience), AnnexinV (88–8103–72, eBioscience), Fixable Viability Dye (FVD) (65-0865-14, eBioscience). Human: CD127 (HIL-7R-M21 BDBiosciences and A019D5, Biolegend), CD4 (OKT4, Biolegend), CD25 (BC96, Biolegend), CD103 (Ber-ACT8, Biolegend), CD45RA (HI100, eBioscience), CD45RO (UCHL1, Biolegend), Foxp3 (259D, Biolegend), CCR4 (TG6/CCR4, Biolegend).

### Quantitative reverse transcription–polymerase chain reaction

T_reg_ cells were sorted by gating on CD4^+^ Foxp3YFP^+^ or CD4^+^CD25^+^ T cells. Tissues were collected in Trizol or RLT buffer (Qiagen RNEasy Kit) and homogenized with the Pro200 Homogenizer (Pro Scientific) and total RNA was extracted. TaqMan one-step RT-PCR (Applied Biosystems, Foster City, CA) or SYBR green q-PCR was performed with a 7500 Real Time PCR System per the instructions of the manufacturer (Applied Biosystems). Expression of the tested genes was normalized to the housekeeping ribosomal protein L19 (rPL19) mRNA. Arbitrary relative expression units were calculated by division of expression of the gene of interest by rPL19 mRNA expression. Primer and probe sequences for each target are available upon request.

### Enzyme-linked immunosorbent assay

Supernatants were taken and used for ELISA. Assay was performed according to manufacturer’s guidelines. TNF-alpha Ready-Set-Go! ELISA kit was purchased from eBioscience. IL-17A and IFN-γ ELISA Max™ Set Standard were purchased from BioLegend.

### Histology

Organs were fixed in 10% formalin for overnight and transferred in to 70% ethanol. Paraffin embedded blocks were sectioned (4–5 μm) and hematoxylin and eosin stained. Slides were scored blindly on a scale of 0–12 if needed, as previously described.

### Statistical analysis

The *p* values for all Figures were calculated with a paired 2-tailed Student’s *t* test. In addition, some Fig. 6 statistics were calculated by one-way ANOVA where indicated. A *p* value <0.05 was considered significant. Error bars denote ±SEM as indicated.

## Electronic supplementary material

Suppemental Figures
