# Supplementary material for: S1P1 deletion differentially affects TH17 and Regulatory T cells
Source: Sci Rep. 2017 Oct 10;7:12905. doi: 10.1038/s41598-017-13376-2 (PMC5635040; doi:10.1038/s41598-017-13376-2)
Supplement: Supplementary file 1 — Suppemental Figures [file 41598_2017_13376_MOESM1_ESM.pdf]

Supplementary Information:

Title: $S1P_1$ deletion differentially affects TH17 and Regulatory T cells

Authors: Ahmet Eken[1,2], Rebekka Duhen[3], Akhilesh K. Singh[1], Mallory Fry[1], Jane H. Buckner[3], Mariko Kita[3], Estelle Bettelli[3,4] and Mohamed Oukka[1,4]

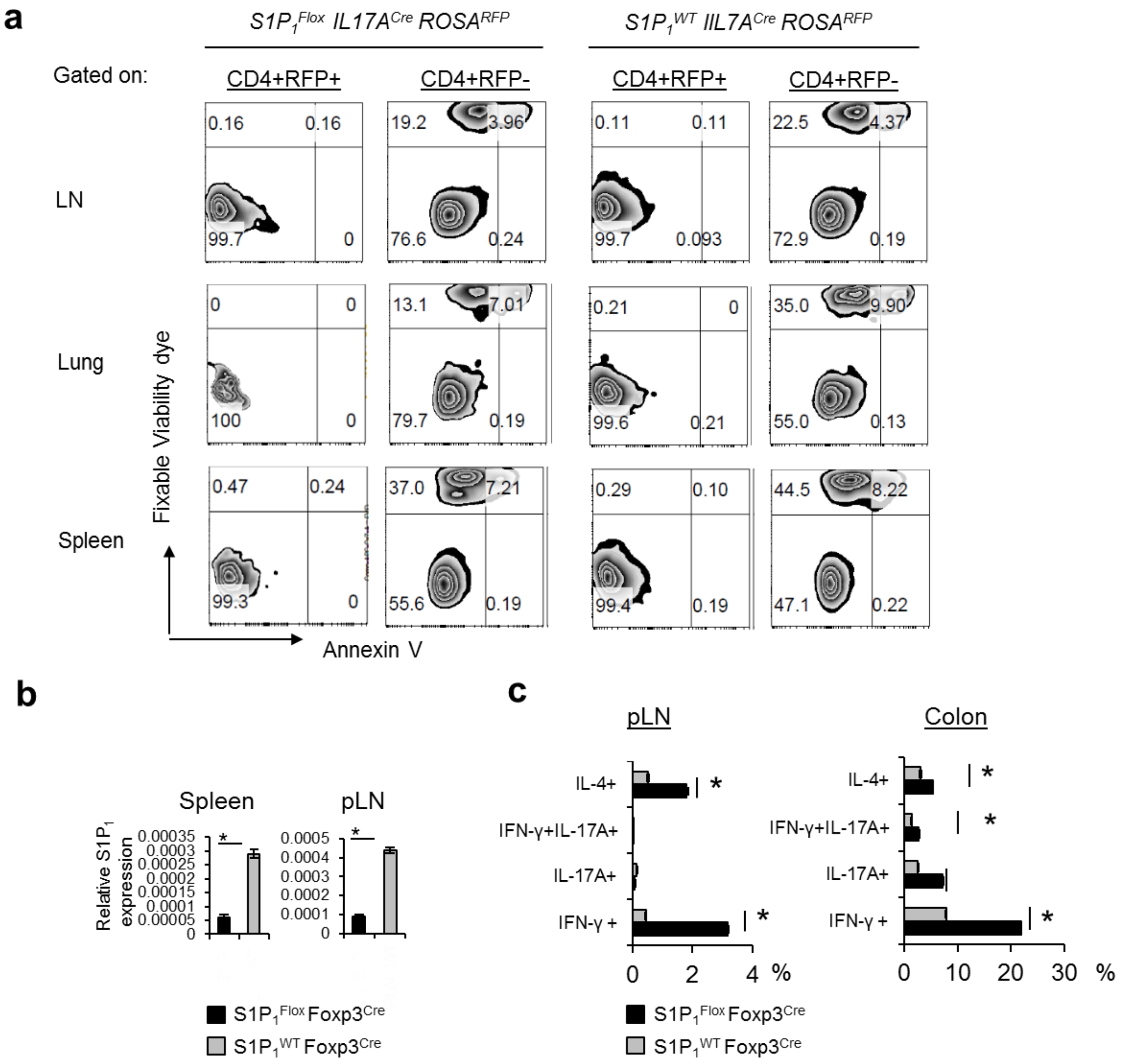

Supplemental Figure 1. **CD4$^+$ T cells are activated in *$S1P_1^{Flox}$ $Foxp3^{Cre}$* mice.** ***Related to Figure 1 and 2***. a) Lymphocytes from LN, Lung and Spleen were cultured overnight in the presence of 20ng/ml IL-23 and apoptosis from indicated 8-week old mice were visualized by Annexin V and fixable Viability Dye staining, CD4$^+$RFP$^+$ or CD4$^+$RFP$^-$ cells were gated. Representative plot is provided. b) T_{reg} cells (CD4$^+$ Foxp3YFP$^+$) sorted from the spleen (SPLN) and peripheral lymph nodes (PLN) of *$S1P_1^{Flox}$ $Foxp3^{Cre}$* and *$S1P_1^{WT}$ $Foxp3^{Cre}$* mice were tested for the expression of $S1P_1$ by qPCR. c) Lymphocytes from LNs or colon lamina propria were prepared and stimulated *ex vivo* with PMA/ Ionomycin for 4 hours and stained for indicated cytokines. CD4$^+$ T cells were gated and percent of cells expressing indicated cytokines analyzed.CD4$^+$ T cells from *$S1P_1^{Flox}$ $Foxp3^{Cre}$* mice express higher levels of IL-4, IFN-γ, and IL-17A. (*) indicates $p<0.05$. (n=3 mice)

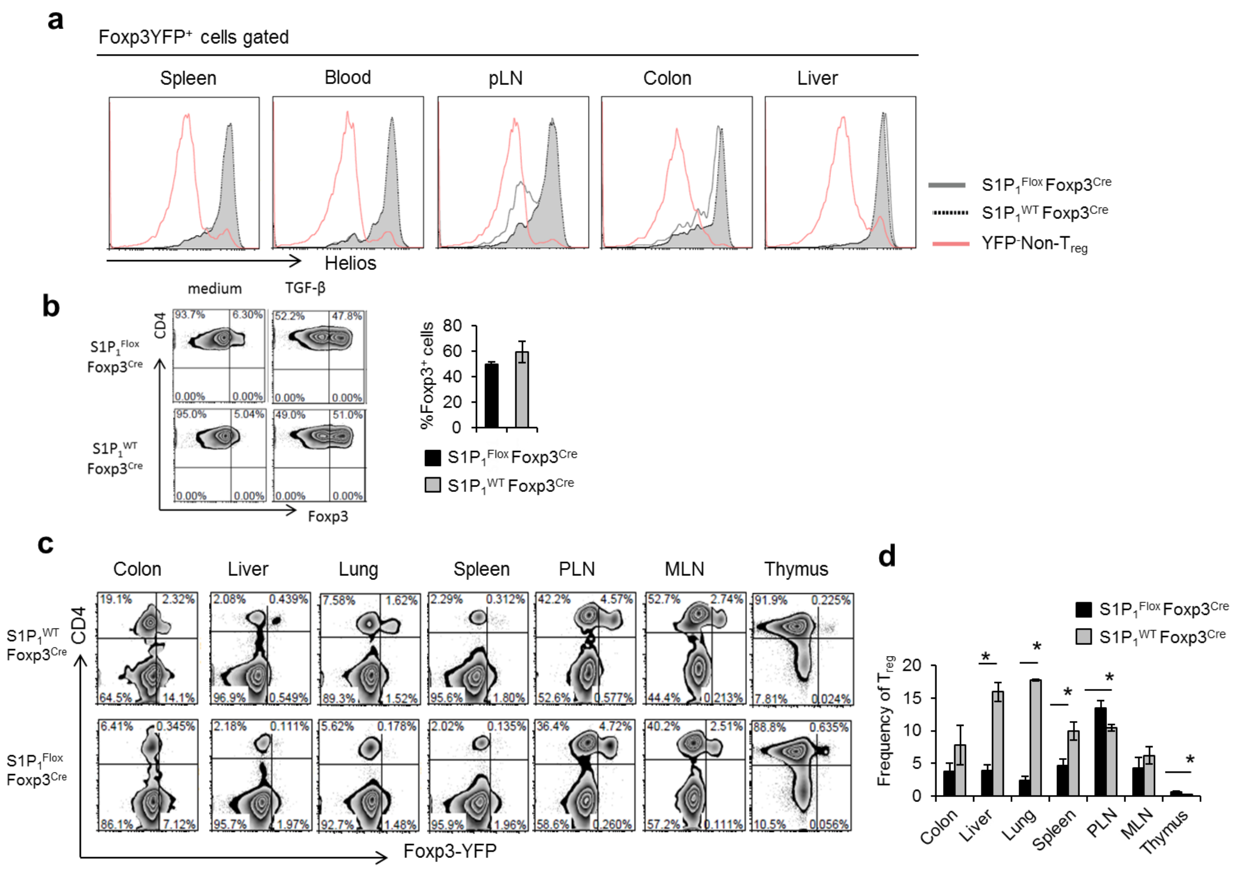

Supplemental Figure 2. Tissue distribution of T_{reg} cells in 7-day-old *$S1P_1^{Flox}$ $Foxp3^{Cre}$* mice and IL-2 responsiveness of $S1P_1$KO T_{reg} cells. *Related to Figure 3*. a) Lymphocytes from indicated organs of 8-week-old WT or *$S1P_1^{Flox}$ $Foxp3^{Cre}$*mice were stained for Foxp3, CD4 and Helios. b) $CD44^{Low}$ $CD62^{high}$Foxp3YFP^{-}naïve CD4^{+} T cells were sorted from 5-week old control or *$S1P_1^{Flox}$ $Foxp3^{Cre}$* mice and *in vitro* differentiated into T_{reg} in the presence of TGF-β, anti-CD3 and irradiated, CD4 depleted splenocytes as antigen presenting cells for 5 days. Foxp3^{+} cells were quantified by flow after nuclear staining. Comparable differentiation into T_{reg} lineage was observed in *$S1P_1^{Flox}$ $Foxp3^{Cre}$* and control mice. c) The distribution of T_{reg} cells (CD4^{+} Foxp3/YFP^{+}) in different organs was determined by staining lymphocytes of 7-day old WT or *$S1P_1^{Flox}$ $Foxp3^{Cre}$* mice with CD4 (representative zebra plot). d) T_{reg} percentages in b are quantified. (*) indicates p<0.05 quantified (e and F).(*) indicated p<0.05. n=5 mice per group.

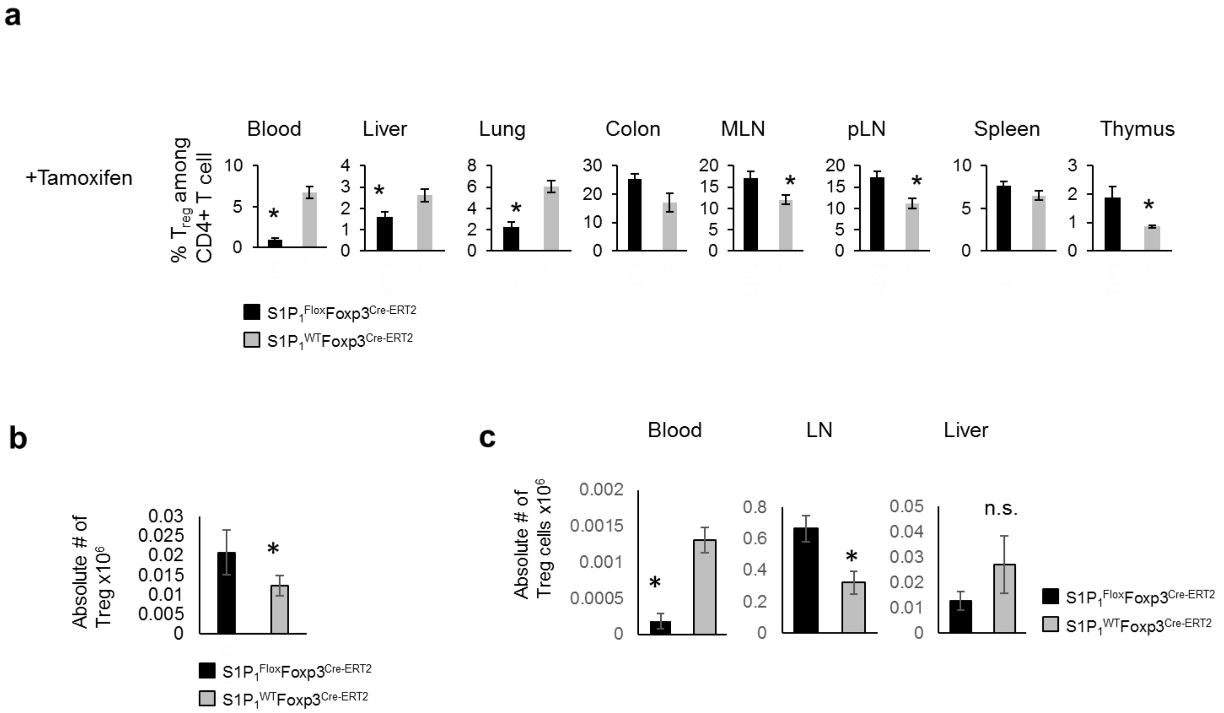

Supplemental Figure 3. Acute deletion of $S1P_1$ in T_{reg} impairs lymphoid tissue egress, reduces non-lymphoid tissue localization and promotes central to effector T_{reg} switch and T_{reg} Function. ***Related to Figure 4.*** a) Lymphocytes from various organs of 6-8-week-old control *$S1P_1^{WT}$ $Foxp3^{Cre-ERT2}$* or *$S1P_1^{Flox}$ $Foxp3^{Cre-ERT2}$* mice were purified on day 7 after 5 daily consecutive tamoxifen injections, and stained for CD4 and Foxp3. T_{reg} distribution was analyzed by flow cytometry and quantified. b) Absolute number of T_{reg} cells in the CNS of *$S1P_1^{Flox}$ $Foxp3^{Cre-ERT2}$* (S1P1KO) and control *$S1P_1^{WT}$ $Foxp3^{Cre-ERT2}$* (WT) mice at the peak of the disease. c) Absolute number of CD4+ Foxp3YFP+ T_{reg} cells in indicated organs purified on day 7 after 5 daily consecutive tamoxifen injections.

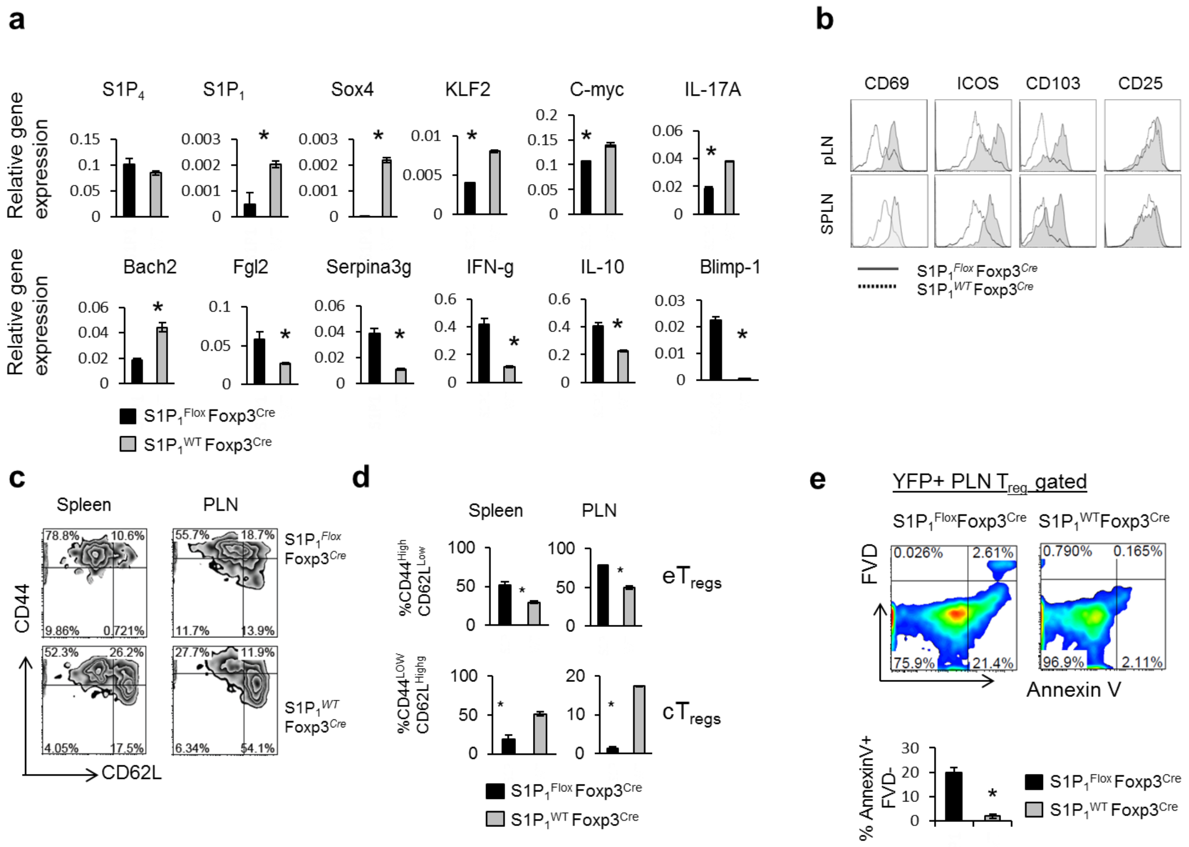

Supplemental Figure 4. Validation of differentially expressed genes in T_{reg} from *$S1P_1^{Flox}$ $Foxp3^{Cre}$* mice by qPCR and phenotypic characterization of T_{reg} cells in *$S1P_1^{Flox}$ $Foxp3^{Cre}$* mice. *Related to Figure 4.* a) Relative expression of indicated genes were determined by real time qPCR performed on T_{reg} cells sorted from the SPLN and PLN of 6 week-old WT or *$S1P_1^{Flox}$ $Foxp3^{Cre}$* mice, n=3 mice per group b) Cell surface expression of CD69, CD103, ICOS and CD25 by splenic T_{reg} cells of 8 week-old WT (*$S1P_1^{WT}$ $Foxp3^{Cre}$)* or *$S1P_1^{Flox}$ $Foxp3^{Cre}$* mice c) Percent eT_{reg} ($CD44^{high}CD62L^{low}$) or cT_{reg} ($CD44^{low}CD62L^{high}$) cells in the spleen of 8 week-old WT (*$S1P_1^{WT}$ $Foxp3^{Cre}$)* or *$S1P_1^{Flox}$ $Foxp3^{Cre}$* mice, n=3 mice per group d) quantification of "c". e) Purified pLN of 8-week-old WT (*$S1P_1^{WT}$ $Foxp3^{Cre}$)* or *$S1P_1^{Flox}$ $Foxp3^{Cre}$* mice were stained for Annexin V and FVD as an indicator of apoptosis n=3 mice per group, representative flow plot (top) and quantification of apoptosis (bottom).

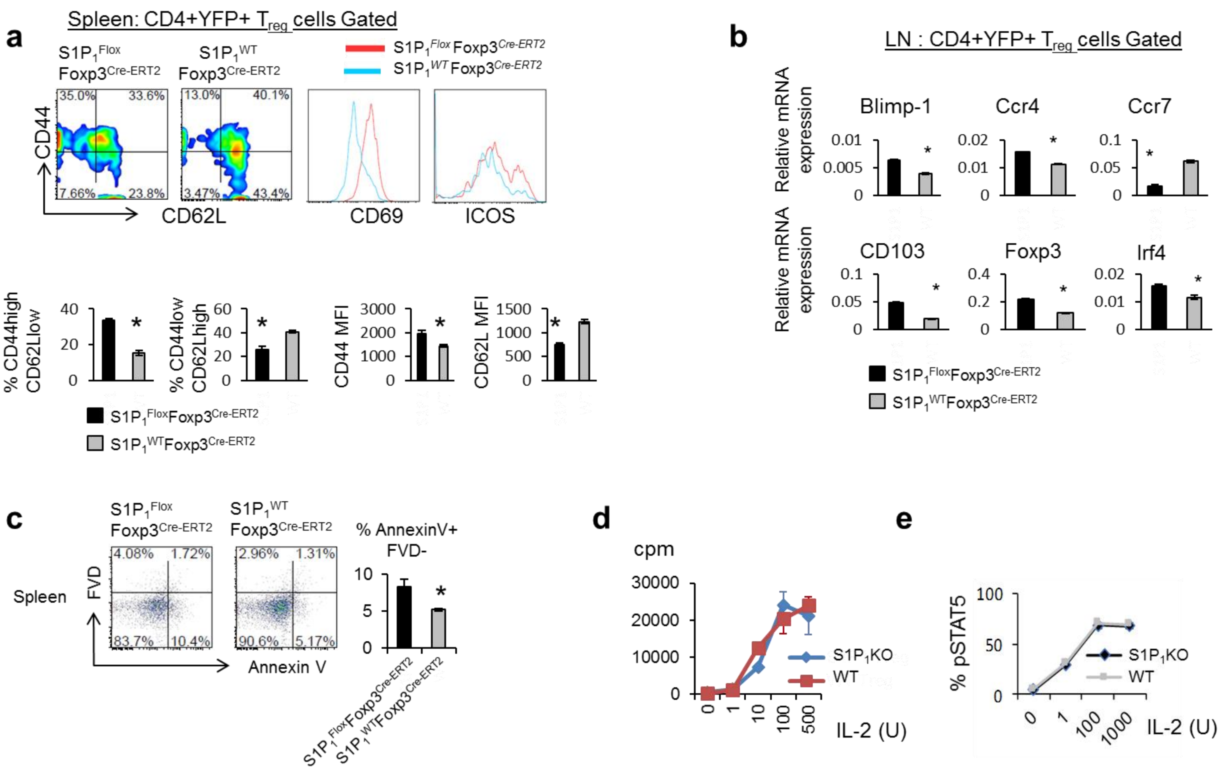

Supplemental Figure 5. Acute deletion of $S1P_1$ in T_{reg} promotes central to effector T_{reg} switch. ***Related to Figure 5.*** a) CD44, CD62L, ICOS and CD69 expression on splenic T_{reg} cells after 5 tamoxifen injections. More eT_{reg} cells were observed in *$S1P_1^{Flox}$ $Foxp3^{Cre\text{-}ERT2}$* mice compared to control mice. (b) Relative gene expression of indicated eT_{reg} associated genes in sorted T_{reg} cells obtained from LN after 5 tamoxifen injections. c) Splenocytes from control or *$S1P_1^{Flox}$ $Foxp3^{Cre\text{-}ERT2}$* mice were stained for Annexin V and viability dye to measure apoptosis after 5 consecutive daily tamoxifen injections. A representative dot plot showing annexin V and viability dye staining in YFP/$Foxp3^+$ $CD4^+$ T cells (left), and its quantification (in 3 mice, on the left) indicate more apoptosis in $S1P_1$KO T_{reg} cells. d) T_{reg} cells were sorted from 6 weeks-old WT or *$S1P_1^{Flox}$ $Foxp3^{Cre}$* mice and co-cultured with irradiated antigen presenting cells and anti-CD3 plus increasing concentrations of IL-2. T_{reg} proliferation was measured by ^{3}H-Thymidine incorporation assay. e) Lymphocytes from LN of 6 weeks-old WT or *$S1P_1^{Flox}$ $Foxp3^{Cre}$* mice were stimulated with indicated concentrations of IL-2 for 20 minutes and STAT5 phosphorylation was quantified by gating $CD4^+YFP^+$ cells.

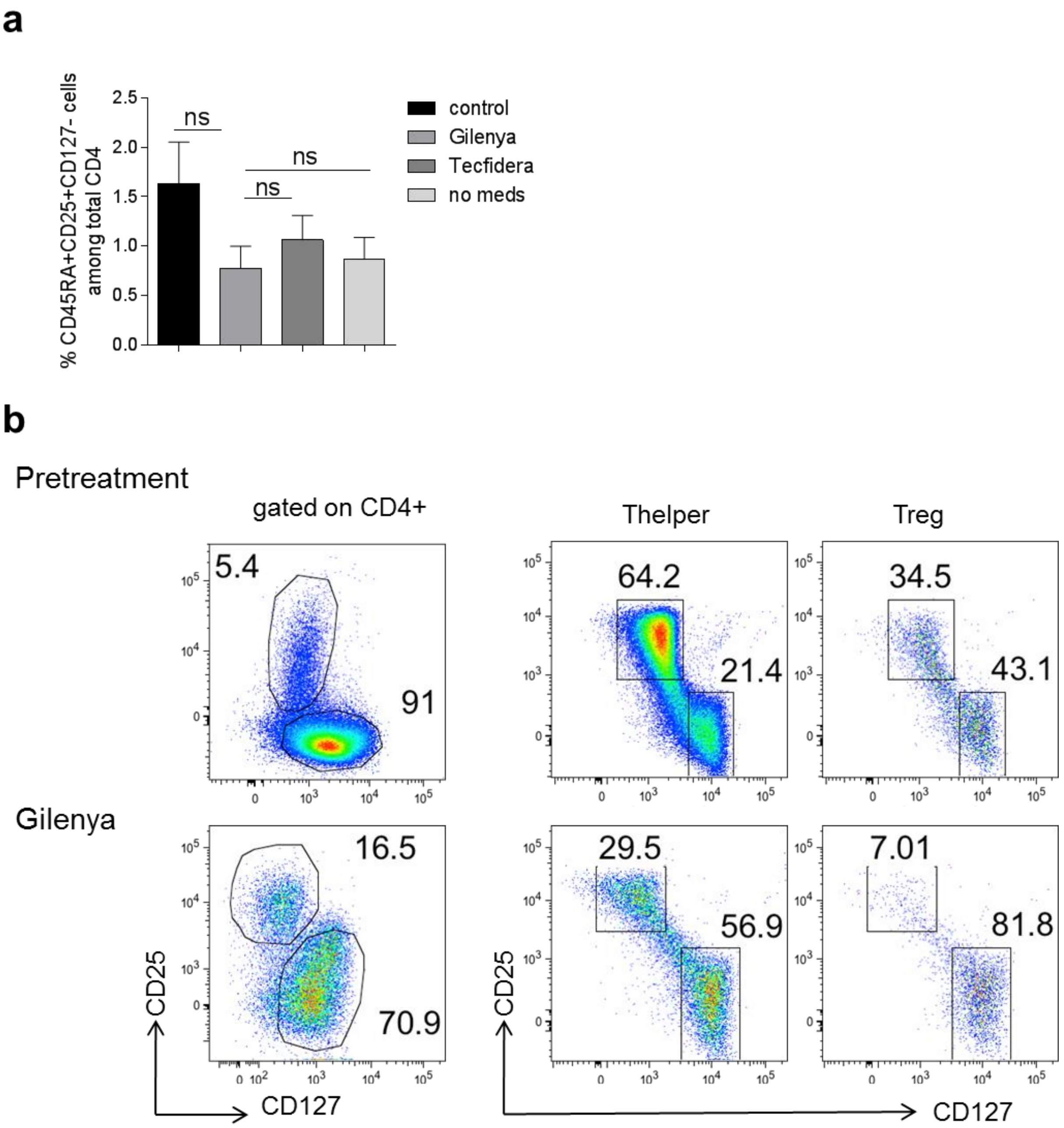

Supplemental Figure 6. **Gating strategy in human PBMC.** ***Related to Figure 6.*** a) Percentage of CD4$^+$CD45RA$^+$CD25$^+$CD127$^-$ Naïve T$_{reg}$ cells in patients treated or not with fingolimod or dimethyl fumarate compared to healthy controls. b) Representative expression of CD25 and CD127 expression by viable CD4$^+$ peripheral blood T cells (*left panels*) and proportion of CD45RA$^+$ and CD45RO$^+$ T helper and T$_{reg}$ cells *(right panels)* from an MS patient before (*prior treatment*) and after treatment with fingolimod (*Fingolimod)*. All data are presented as frequency among total CD4$^+$ T cells.
